# Natural supplements in breast cancer therapy: translational evidence, clinical safety, and emerging challenges

**DOI:** 10.3389/fonc.2026.1748136

**Published:** 2026-04-30

**Authors:** Sirajunisa Talath, Mohamed El-Tanani, Adil Farooq Wali, Syed Arman Rabbani, Bhoomendra Bhongande, Walaa Ibraheem, Ashot Avagimyan, Karolina Hoffmann, Ioannis Ilias, Sorina Ispas, Maggio Viviana, Anna Paczkowska, Manfredi Rizzo

**Affiliations:** 1 Ras Al Khaimah (RAK) College of Pharmacy, Ras Al Khaimah (RAK) Medical and Health Sciences University, Ras Al Khaimah, United Arab Emirates; 2 Department of Internal Diseases Propedeutics, Yerevan State Medical University after M. Heratsi, Yerevan, Armenia; 3 Isfahan Cardiovascular Research Centre, Cardiovascular Research Institute, Isfahan University of Medical Sciences, Isfahan, Iran; 4 Department and Clinic of Internal Diseases and Metabolic Disorders, Poznan University of Medical Sciences, Poznań, Poland; 5 Department of Endocrinology, Diabetes and Metabolism, Elena Venizelou Hospital, Athens, Greece; 6 Department of Anatomy, Faculty of General Medicine, “Ovidius” University, Constanta, Romania; 7 School of Medicine, Department of Health Promotion, Maternal and Infant Care, Internal Medicine and Medical Specialties (PROMISE), University of Palermo, Palermo, Italy; 8 Department of Pharmacoeconomics and Social Pharmacy, Poznan University of Medical Sciences, Poznań, Poland

**Keywords:** breast cancer, curcumin, luteolin, natural supplements, quercetin, resveratrol, withaferin A

## Abstract

The incidence and mortality rates of breast cancer are increasing, although improvements in traditional treatments, and it remains the most frequent cancer among women globally. The increasing demand for natural supplements and other forms of complementary and integrative medicine (CIM) shows that people are becoming increasingly intrigued by more comprehensive methods of health care that aim to improve people’s well-being as well as their ability to recover from illness. The objective of this review article is to focus on the clinical safety features supported by data and the translational utility of the natural supplements such as vitamins, minerals and phytochemicals against breast cancer. Anticancer activity by antioxidants, anti-inflammatory agents, apoptotic inductors, and hormonal modulators characterize the behaviour of such natural compounds as curcumin, withaferin A, luteolin, quercetin, and resveratrol. Preclinical and early-phase clinical studies suggest that certain natural supplements may modulate therapeutic response and influence treatment-related toxicity; however, robust clinical evidence remains limited, and definitive conclusions regarding efficacy require further well-designed randomized trials Indeed, the lack of bioavailability, standardization, and the risk of herb–drug interaction prevent these agents from becoming the prevailing therapy option. However, several well-functioning strategies that promote the development of nanotechnology-based formulations and corresponding optimal doses appear to be promising. Even though the regular administration of natural supplements can have significant potential within breast cancer management, the process should be consistent with the principles of evidence-based practice. At the same time, there is the need for clinical trials focused on the development of corresponding pharmacovigilance systems and closer interdisciplinary collaboration. Importantly, future studies are expected to make special emphasis on such factors as dosing personalization, standardization parameters, and their integration with precision oncology.

## 1 Introduction

Breast cancer remains a significant public health problem because to its high incidence, extensive morbidity, or fatality rates, and the fact that it is the most frequent cancer in women worldwide, even though screening and therapy have made advances (1). The estimated death rate is expected to rise, and the number of new cases is expected to surpass half a million by 2040. Breast cancer accounts for 16% of all cancer-related fatalities and 1 in 4 cases of cancer overall (2). Over half a million cases will have been reported by 2040, and the mortality rate will have increased by the same amount (3). At 16.7 percent of the diagnoses of cancer & cancer-related deaths worldwide, breast cancer remains supreme (4). There are an estimated 2.30 million annual diagnoses of breast cancer, according to recent statistics released by the American Cancer Society (ACS).

Factors such as increased life expectancy, more urbanisation, different lifestyle choices, and eventual reproductive ages are contributing to the disease’s increasing prevalence, particularly in countries with low or middle incomes (LMICs) (5). A significant amount of molecular, genetic, and clinical variability is observed among the illness’s subtypes, which include hormone receptor-positive (HR-positive), enriched in HER2, or triple-negative breast cancer (TNBC). These subtypes display varied risk profiles, treatment responses, and prognostic behaviours. Factors that have been identified as potential causes of breast and ovarian cancer include advanced age, a personal or family history of the disease, a positive or negative BRCA1/BRCA2 mutation status, hormonal changes (during menstruation and after menopause), the use of oral contraceptives or HRT, excess body fat, alcohol consumption, lack of physical activity, and environmental carcinogens (6, 7).

The upsurge of interest on natural supplements such as phytochemicals, plant extracts, and bioactive components (namely vitamins, minerals, polyphenols, flavonoids and nutraceuticals) for their potential anticarcinogenic, antioxidant, anti-inflammatory as well as immune-modulatory effects over and above conventional breast cancer management has been observed in response to the aforementioned clinical challenge. The medicinal benefits of herbal medicine in cancer treatment have been highlighted in published research (8, 9). Traditional medical systems, including Ayurveda and TCM, rely heavily on medicinal herbs. A wide range of pharmacologic actions, such as antioxidant, anti-inflammatory, anticancer, immunologic modulatory, and apoptotic properties, are exhibited by the bioactive components that make up herbal medicines (10–12).

However, the standardisation of herb-derived products, safety concerns regarding chronic consumption or concurrent use with traditional chemotherapeutics, inadequate large-scale randomised controlled trials, low product bioavailability, and herb-drug interactions have all impeded the clinical translation of natural interventions. This highlights the urgent need for strong clinical evidence, aligned regulatory networks and integrated research strategies to fill the gap between such exciting preclinic findings and their translation into patient outcomes. The changing field of translational oncology highlights the need for multidisciplinary teamwork including clinicians, pharmacologists, toxicologist and traditional medicine scientist in development of evidence based guidelines towards safe and efficient use of natural supplements alongside breast cancer therapy.

The review is unique in that it provides an exhaustive overview of nutraceutical-pharmacological integration in breast cancer. Specifically, the researchers discuss the molecular mechanisms of the anticancer action of all major natural supplements implicated in the condition, while simultaneously focusing on the peculiarities of their synergistic interaction with hormonal agents and conventional chemotherapeutics. Concurrently, the review places a special emphasis on the clinical validation status of the selected combinations, including at the expense of discussing the most prevalent pharmacokinetic problems, formulation issues, and patient safety concerns.

### 1.1 Role of complementary and integrative medicine in breast cancer

Conventional therapies have improved our understanding of the disease and increased survival rates, but they may not be able to address the complete range of physiological, psychological, or quality-of-life needs that breast cancer patients may have, which is why complementary or integrative medicine (CIM) has emerged as a vital component of overall breast cancer treatment (13, 14). Natural supplements, as one of the most commonly used parts of CIM in breast cancer, have attracted attention because they may perform anticarcinogenic, antioxidant, anti-inflammatory, immunomodulatory and antiangiogenic activities and pro-apoptotic functions; they could thus provide a promising adjuvant to surgery, chemotherapy/anthracycline/taxane/radiotherapy/targeted/endocrine therapy (15).

Widely studied supplements include curcumin, green tea catechins (EGCG), resveratrol, quercetin, genistein (soy isoflavones), omega-3 fatty acids, silymarin, ashwagandha and vitamins D and selenium as well as some of the mushrooms such as Ganoderma lucidum many of which have preclinical evidence for inhibiting tumour proliferation, chemosensitizing cancer cells modulating oestrogen receptor signalling decreasing oxidative stress or stimulating immune surveillance (16, 17).

Unlike prior reviews that primarily summarize bioactive compounds, the present manuscript adopts an integrative translational framework that combines molecular mechanisms, clinical evidence, safety evaluation, pharmacokinetic considerations, nano-delivery innovations, and precision oncology perspectives. The objective is to bridge experimental findings with responsible clinical implementation and to clarify the therapeutic potential and limitations of natural supplements in contemporary breast cancer management.

### 1.2 Distinctive contribution of the present review

Several previously published reviews have summarized the biological activities of natural compounds and dietary supplements in breast cancer. However, many of these articles primarily provide descriptive overviews of phytochemicals or focus predominantly on preclinical mechanistic findings without systematically integrating translational, clinical, pharmacokinetic, and safety dimensions within a single framework.

The present review differs in scope and structure by adopting a translational oncology perspective that bridges molecular mechanisms, clinical trial evidence, therapeutic interactions, and real-world safety considerations. Rather than cataloguing compounds individually, this manuscript synthesizes mechanistic pathways with available human data and evaluates their relevance across breast cancer subtypes, including hormone receptor–positive, HER2-positive, and triple-negative disease. In addition, it incorporates discussion of bioavailability limitations and emerging nanotechnology-based delivery systems designed to enhance clinical applicability.

Importantly, this review provides an expanded evaluation of drug–supplement interactions, including cytochrome P450–mediated metabolic interference, potential modulation of endocrine therapy metabolism, effects on chemotherapy-induced oxidative stress mechanisms, and considerations relevant to immunotherapy. Furthermore, precision oncology concepts—such as host genetic variability, microbiome–oestrogen interactions, and subtype-specific responsiveness—are integrated to contextualize individualized supplementation strategies.

By consolidating mechanistic insight, clinical validation, safety profiling, pharmacokinetic challenges, and translational strategies into a unified model, this manuscript aims to provide an evidence-based and clinically actionable framework that extends beyond prior narrative summaries.

## 2 Clinical evidence for natural supplements

### 2.1 Vitamins and minerals

Researchers have been a lot of interest in the clinical evidence linking vitamins and minerals, especially vitamin D, calcium, and selenium, to the treatment and prevention of breast cancer (18). One of the most researched micronutrients, vitamin D, exerts its effects via a vitamin D receptor (VDR) that represses tumour angiogenesis and proliferation, as well as genes involved in cell differentiation, immunological regulation, and apoptosis (19). Evidence from observational cohorts suggests that women with lower vitamin D levels are more likely to develop aggressive tumour subtypes, such as triple-negative breast cancer (TNBC), and have inferior survival rates (20). Numerous epidemiological investigations have shown a negative correlation between serum 25-hydroxyvitamin D [25(OH)D] and breast cancer development and death. It has been found in large population-based research that elevating 25(OH) D levels above 30–40 ng/mL is often linked to a decreased risk of disease progression and better overall survival. However, RCTs have shown contradictory findings, which is frequently attributable to variations in baseline vitamin D levels, dosage, and follow-up duration across studies (21). Furthermore, the benefits of vitamin D supplementation on endocrine-based therapies have also been reported in smaller intervention studies where tamoxifen–mediated oestrogen receptor signalling and aromatase expression decreases were observed, as well as a more general protective role against bone demineralization often seen in long-term anti-oestrogen therapy patients (22).

Patients with breast cancer, whether they utilise aromatase inhibitors or are undergoing chemotherapeutic, ovarian failure, which promotes bone loss and increases the risk of fractures, must have adequate calcium and vitamin D levels checked regularly to keep their bones healthy (23–25). The calmodulin-dependent signalling pathways have led researchers to believe that calcium regulates cell proliferation and death in addition to its effects on bone. Despite the lack of convincing evidence for a direct anticancer effect of calcium alone, clinical trials evaluating supplementation with vitamin D have primarily reported on bone mineral density (BMD) retention and prevention of skeletal events in breast cancer survivors. This suggests that combining the two can significantly reduce the risk of fracture and keep bones strong (26, 27).

### 2.2 Phytochemicals

#### 2.2.1 Curcumin

Curcumin, the principal physiologically active polyphenolic substance in *Curcuma longa* (turmeric) (28), is one of the most studied herbal substances because of its wide range of anticancer effects. According to preclinical research, curcumin can hinder tumour growth by blocking pathways like NF-κB and STAT3, reducing IL-6 and TNF-α levels, altering the expression of apoptotic regulators like Bcl-2 and Bax, preventing angiogenesis through Vascular Endothelial Growth Factor (VEGF) suppression, and influencing the signalling of HER2 and oestrogen receptors (29). In hormone receptor-positive or triple-negative breast cancer cell lines, curcumin may be able to reverse multidrug resistance by preventing efflux transporters like P-glycoprotein, radiosensitize cells by regulating oxidative stress, and activate cells towards chemotherapeutic drugs like paclitaxel or doxorubicin (30).

Despite the small sample sizes and dosages used in these clinical trials, the mounting evidence suggests that oral curcumin is well-tolerated, decreases markers of systemic inflammation, protects against mucositis caused by chemotherapy, and alleviates pain in the joints in patients taking aromatase inhibitors (31). However, the low bioavailability, rapid metabolism and poor tissue accumulation of curcumin continue to be major obstacles to reaching therapeutic levels with oral dosing thereby driving further research on nanoformulations along with liposomal formulations of curcumin and complexes such as curcumin-phospholipid (e.g., Meriva^®^) for improved bioefficacy.

Researcher synthesised demethoxycurcumin and bis-demethoxycurcumin, in HERr2 + cells and MCF-7, the phytochemicals were discovered to limit cancer cell proliferation, as shown by an IC50 value below 10 μM (32). It should be noted that *in vitro* IC_50_ concentrations may not directly reflect clinically achievable plasma or tissue levels, and translational interpretation requires pharmacokinetic validation. In MCF-7 cells, it induced apoptosis and blocked cell cycle progression, hence mediating cell death (33). Fuchs et al. synthesised a class of curcumin analogues called the pentadienones (34). Against MCF-7 and MDA-MB-231 cells, these compounds showed substantial antiproliferative effect with IC50 values of 0.40 μM and 0.60 μM, respectively. It should be noted that *in vitro* IC_50_ concentrations may not directly reflect clinically achievable plasma or tissue levels, and translational interpretation requires pharmacokinetic validation. Curcumin is an active component in breast cancer therapy, and these forms make it much more bioavailable.

According to research, curcumin has the potential to slow the progression of breast cancer by controlling the expression of flap endonuclease 1 via the NRF2 signalling pathway (35). After being injected intravenously with curcumin, breast cancer mice show a marked decrease in tumour growth and metastasis (36). Breast cancer patients who have established a resistance to hormonal treatment may be able to reverse it by mixing tamoxifen with curcumin or by taking it alone. Apoptosis induction and inhibition of cancer cell growth are achieved by interacting with different signalling pathways that are associated with resistance or survival (37). Curcumin and docetaxel, when administered together, have the same anti-cancer and slowing effects on tumour indicators as each chemical alone, according to clinical trials (38).

One study found that curcumin-dovetaxel co-delivery liposomes (CUR-DTX-L) had better anticancer activity and better pharmacokinetic parameters (longer half-life, mean residence duration, etc.) than drug-free medicines in MCF-7 breast cancer xenograft models (39). Concurrently, SLN-loaded with curcumin exhibited enhanced cellular uptake and cytotoxicity towards breast cancer cells (40). These nanoparticles enhanced apoptotic rates in comparison to free curcumin, suggesting they could be valuable chemotherapeutic formulations (41). A more targeted delivery of curcumin to breast cancer cells has been achieved through the use of derivatives like RGD peptide-anchored liposomes. These FADs significantly enhanced cytotoxicity and apoptosis in MCF-7 cells when contrasted with non-targeted FADs (42). In contrast to free medications, the CUR-DTX-L cluster enhanced curcumin’s pharmacokinetics, which in turn improved its plasma concentration- time curve, average residence duration, and biological half-life. A longer duration of action and a more progressive release of curcumin are suggested by this enhanced profile (43). Research suggests that SLNs greatly enhance the therapeutic effectiveness of curcumin by altering its release kinetics and increasing its blood circulation (44).

#### 2.2.2 Withaferin A

Withaferin A (WA) bioactive compound found in Ashwagandha (*Withania somnifera*) has been shown in numerous *in vitro* and *in vivo* studies to have potent anti-cancer properties (45). By blocking aerobic glycolysis in breast cancer cells, WA was able to exert its anticancer effects. Researcher suppressed a large number of glycolytic enzymes by focussing on the c-MYC pathway. This pertains to the following enzymes glucose transporter 1, pyruvate kinase muscle isozyme 2, and hexokinase 2. Cell survival and tumourogenicity were reduced by the inhibition of glucose absorption, lactate production, and ATP creation (46). In addition, it was discovered that WA diminished the effects of TNFα and TGFβ on non-tumourigenic MCF-10A cells during EMT as altered EMT markers such as E-cadherin and vimentin in breast cancer cell lines.

Xenograft and transgenic mouse tumour models showed anticancer effectiveness after WA downregulated the vimentin expression (47). In contrast to its inert effect on normal mammary epithelium cells, WA generated ROS and caused cell death in the MDA-MB-231 and MCF-7 breast cancer lines. One mechanism by which WA induces cell death is by inhibiting mitochondrial complex III, which is involved in oxidative phosphorylation (48). Mitochondrial DNA activates the B-cell lymphoma 2 (BCL-2) antagonist/killer signalling pathway to achieve this condition. According to another study, WA’s anticancer effects are partially caused by its ability to manipulate the p53 and ERα pathways, which in turn induce human breast cancer cells to go through programmed cell death (49). These results offer new perspective on WA’s cancer war, especially its approach to breast cancer. Cancer cells were rendered more susceptible to proapoptotic agents such as celecoxib, etoposide, and TRAIL when WA activated the ETS-like transcription factor 1/C-EBP identical protein pathway and the extracellular signal-regulated kinase (ERK) ribosomal S6 kinase signalling pathway, leading to the expression of death receptor 5. Xenograft & MMTV-neu models shown that WA considerably inhibited the *in vivo* growth of breast cancers (50).

#### 2.2.3 Luteolin

Luteolin first came from the leaves of the *Reseda luteola* plant. It is naturally present in peppers, honeysuckle, chrysanthemums, broccoli, and Brussels sprouts, and it is also present in wine and other edible items (51). Recent advances in luteolin extraction technology have allowed for more efficient processes, such as ultrasonic-assisted acetone extraction for high yields (52) and olive maceration with ethanol and sodium metabisulfite. It has activities against inflammation, cancer, and allergies by suppressing NF-κB, which decreases tyrosine phosphorylation, gene expression, and cytokines (such as IL-6 and TNF-α) (53). Dai et al. found that lutein successfully inhibits tumour growth *in vivo* or EMT, migration of TNBC cells (54). The therapeutic effects of luteolin are enhanced when used in conjunction with other medications. Its capacity to increase the sensitivity of HER2+ breast cancer cells when given with lapatinib is one example. In addition, it suppresses the proliferation of various breast cancer lineages when coupled with celecoxib (55).

Combining celecoxib and luteolin reduced cell proliferation in SkBr3 and MDA-MB-231 cells, while celecoxib suppressed AKT activity and luteolin boosted the ERK signalling pathway in MCF-7 cells (56). Cancer cells that were ER-positive and resistant to tamoxifen were more likely to undergo cell death when treated with a combination of luteolin and inhibitors of PI3K, AKT, or mTOR (57). Treatment with low doses of luteolin reduces doxorubicin toxicity in MCF-7 cells by increasing Bcl-2 expression. The anti-angiogenic properties of luteolin make it a powerful cancer fighter. It stopped angiogenesis and tumour growth in mouse breast cancer xenografts by reducing VEGF levels. Cancer prevention on 4T1 and MDA-MB-231 was improved by combining 40 μM luteolin and doxorubicin. This was achieved by reducing colony formation and invasion, or by activating the cascade of Bax/Bcl-2/Caspase-3 (58).

#### 2.2.4 Ginseng

Ginseng (*Panax ginseng* and *Panax quinquefolius*) is another popular botanical extract in the field of complementary oncology and contains compounds, so-called ginsenosides with pharmacologic activities that are adaptogenic, antioxidant and immunomodulatory and these effects could possibly be beneficial in cancer chemoprevention and therapy (59). According to preclinical research, Rg3 and Rh2 have antitumour effects by reducing tumour growth, anti-angiogenesis, metastasis potential, and drug resistance in both laboratory and animal studies. These effects are achieved by suppressing tumour growth, inducing apoptosis via activation of caspase 3, anti-angiogenesis through VEGF inhibition, and sensitising several chemotherapeutic agents to both *in vitro* and *in vivo* drug resistance mechanisms (60). Because of its immune enhancing and anti-inflammatory characteristics, ginseng has been proven in observational studies to improve overall survival and lower the chance of breast cancer recurrence (61).

#### 2.2.5 Resveratrol

**Figure 1:**
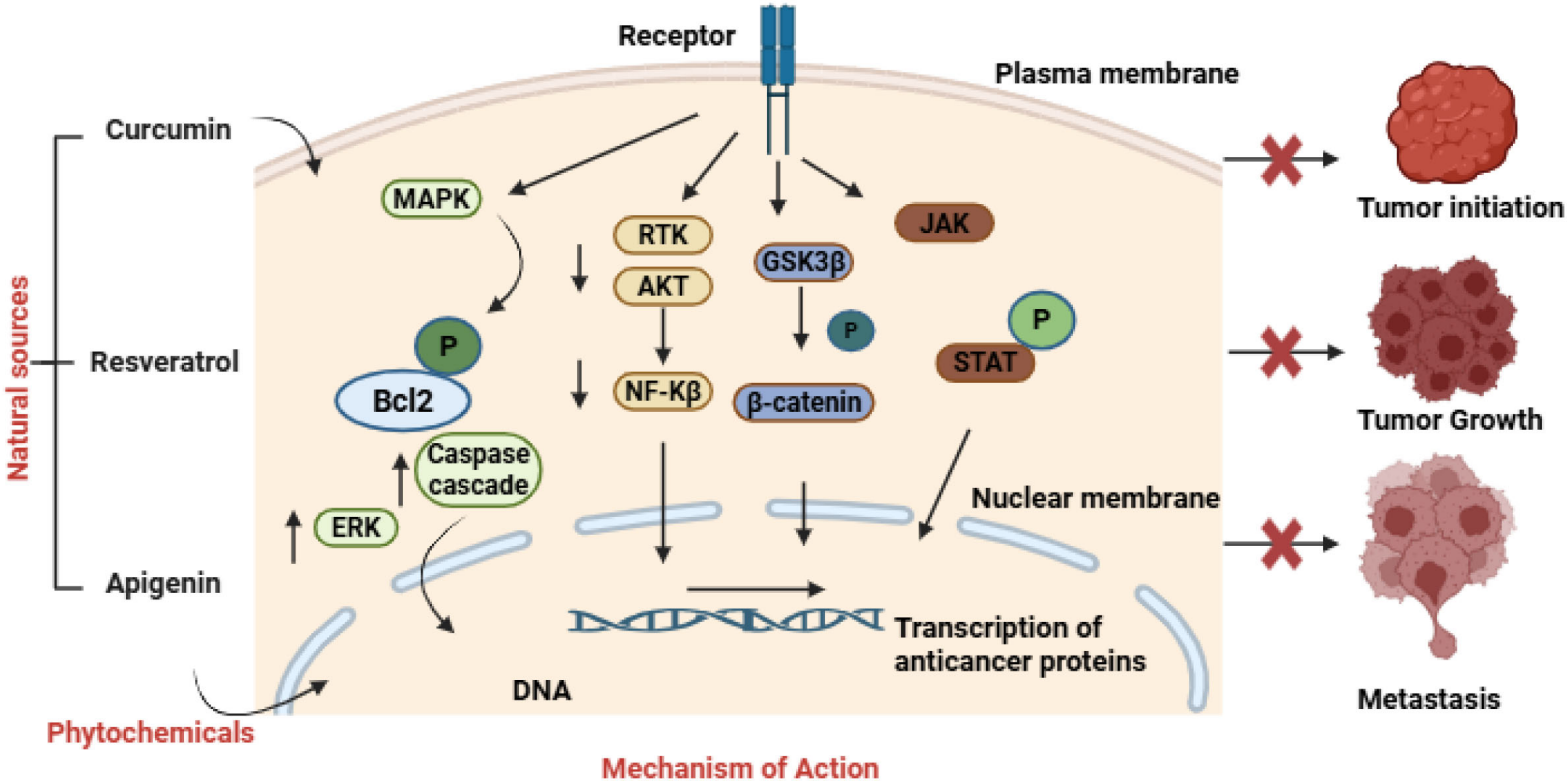
Molecular basis of action of phytochemicals in breast cancer. Created in BioRender. https://app.biorender.com/illustrations/68ef2fef7325c5bb0ffecea4. Schematic showing the mechanistic pathways by which natural phytochemicals Curcumin, Resveratrol and Apigenin possess anticancer activity in breast cancer. They affect fundamental signaling pathways such as MAPK, AKT, ERK, JAK/STAT, and NF-κB that are often dysregulated in breast tumors. During the entry into the cell through receptor mediated method, they regulate apoptotic regulators like Bcl2 and activate the caspase cascade to induce cell death. Furthermore, they also regulate GSK3β and β-catenin to inhibit Wnt signaling and oncogenic transcription. Inside the nucleus, these phytochemicals increase transcription of anticancer proteins and decrease transcription of tumor-promoting gene expression. The sum effect of above interactions ultimately leads to tumor initiation inhibition, tumor growth suppression and metastasis prevention.

Grapes, peanuts, chocolate, berries, red wine, and many other foods contain the natural polyphenol resveratrol, whose chemical name is trans-3,4,5-trihydroxystilbene. Among its various biological advantages are its ability to combat cancer, lower inflammation, and keep the heart and neurological system healthy (62). By blocking the RhoA/Lats1/YAP signalling pathway, resveratrol reduced the invasion of breast cancer cells (**Figure 1**). Beginning with the inhibition of RhoA and continuing via the activation of Lats1 kinase, resveratrol reduced cell proliferation & invasion by phosphorylating and making inactive a critical transcriptional coactivator. Reducing the proliferation of breast cancer cells by blocking this cascade, which was accomplished by lowering the expression levels of YAP gene targets (63). Resveratrol inhibited fibroblast migration, invasion, and enhanced stemness in breast cancer cells, according to the study’s authors.

The antioxidant resveratrol repressed SRY-box 2, blocked AKT, and inhibited signal transducer and activator of transcription 3 signalling, among other effects. It also downregulated important oncogenic proteins including c-MYC, Cyclin D1, MMP-2, and MMP-9. Based on these findings, resveratrol may have therapeutic potential by targeting the tumour microenvironment, as it prevents tumour cells from interacting with cancer-associated fibroblasts (64). Resveratrol considerably reduced TNBC cell growth by lowering the expression of the DNA polymerase delta catalytic subunit (POLD1), a gene that is involved in DNA replication and repair. The result provoked cell death, as evidenced by the increased production of cleaved caspase 3 & cleaved poly(ADP-ribose) polymerase 1. A related study found that resveratrol inhibited oestrogen-induced breast carcinogenesis by activating a protective signalling pathway mediated by nuclear factor kappa B2 (NF-κB2) (65).

#### 2.2.6 Quercetin

Numerous foods include the plant flavonoid quercetin, which has been the topic of substantial investigation; these foods include broccoli, apples, kale, and onions (66). Past and present techniques, such as enzyme-assisted extraction (EAE), supercritical fluid extraction (SFE), and ultrasonic-assisted extraction (UAE), have been employed to maximise its extraction (67, 68). Inhibiting oncogenic kinases and modulating associated signalling pathways make up the bulk of quercetin’s anticancer actions. Quercetin induced apoptosis in TNBC cells via decreasing FASN and β-catenin levels. The p38 MAPK pathway was also suppressed, leading to increased MCF-7 apoptosis. Quercetin promoted miR-146a expression, which quenched the *in vivo* breast cancer cell proliferation (69).

Quercetin inhibits the TNBC-extended pathway in addition to the EMT markers (vimentin, cyclin D 1, c-Myc, and β-catenin) (70). As a key component of the angiogenesis pathway, quercetin may reduce the proliferation of breast cancer xenografts (71). When applied to breast cancer, quercetin either overcomes treatment resistance or complements other anti-cancer drugs. Increasing quercetin’s bioavailability might be achieved by changing its molecular structure (72). A 48-hour treatment with conjugates either one or three times of gold nanoparticles in quercetin considerably decreased the viability of MCF-7 and MDA-MB-231 cells, respectively, with IC50 values of 30.8 μM and 100 μM. They also prevented MCF-7 and MDA-MB-231 from growing by turning off the EGFR signalling pathway (73). Nanoparticle drug carriers enable the efficient administration of all-natural components in the fight against breast cancer (**Table 1**).

**Table 1:** Nanotechnology-based formulations for enhanced bioavailability.

Formulation type	Phytochemical	Particle size (nm)	Tumour inhibition	Reference
Liposomal Curcumin	Curcumin	163	Improved solubility, prolonged circulation, enhanced tumour targeting	(74)
Solid Lipid Nanoparticles	Curcumin	208	Increased cellular uptake, higher apoptosis, sustained release	(75)
Co-delivery Liposomes	Curcumin + Docetaxel	175	Synergistic cytotoxicity, enhanced pharmacokinetics	(43)
Gold Nanoparticle Conjugates	Quercetin	–	EGFR inhibition, reduced cell viability, improved bioavailability	(76)
Nanostructured Lipid Carriers	Resveratrol	125	Enhanced stability, better tissue penetration	(77)
PEGylated Nanoparticles	Withaferin A	25.60	Reduced systemic toxicity, improved tumour accumulation	(78)
Micellar Formulation	Luteolin	100	Enhanced solubility, improved oral absorption	(79)
Nanoemulsion	Genistein	154	Improved permeability, hormone receptor modulation	(80)
Polymeric Nanoparticles	EGCG	67	Controlled release, reduced degradation	(81)
Liposomal Silymarin	Silymarin	–	Liver protection, enhanced antioxidant delivery	(82)
Berberine Nanoparticles	Berberine	191	AMPK activation, improved cytotoxicity	(83)
Thymoquinone Lipid Carriers	Thymoquinone	–	Enhanced ROS generation, better tumour penetration	(84)
Astragalus Nanoparticles	Astragalus polysaccharide	–	Immunomodulation, improved chemo tolerance	(85)
Boswellic Acid Nanoparticles	Boswellic acid	–	Anti-inflammatory delivery, improved bioefficacy	(86)
Honokiol Nanoparticles	Honokiol	–	ERK inhibition, neuroprotective adjunct	(87)

### 2.3 Medicinal plants

#### 2.3.1 *Scutellaria baicalensis* (Chinese skullcap)

A traditional Chinese plant known as *Scutellaria baicalensis* (Chinese skullcap; Lamiaceae) has a long history of medicinal use in East Asian countries (88). *Scutellaria baicalensis* inhibits transcription-regulating genes and mediates phosphorylating kinases in cellular signalling pathways are two of its anti-breast cancer actions (89). *Scutellaria baicalensis* contains two bioactive flavonoids, baicalin and baicalein, which have shown potential in the treatment of breast cancer (90). Baicalin inhibits the migration of highly mobile breast cancer cells, specifically MDA-MB-231, via binding to β-catenin signalling and reversing EMT (91). Inducing apoptosis in MCF-7 cells through down-regulating BCL-2 and up-regulating BAX and p53 via activation ERK/p38-mediated caspase 9/3 signalling was possible with baicalin coupled with baicalein (92). By utilising the BCL−2 and BAK signalling pathway, which includes caspase, and boosting ROS, Park et al. discovered that SBGE inhibited cell growth and enhanced apoptosis. Also, pretreatment with MAPK & c-jun N-terminal kinase inhibitors exhibited an inhibitory effect on death, suggesting that the MAPK pathway was the medium via which the SBGE’s apoptotic effects passed (93).

Different research found that while the full *Scutellaria baicalensis* extract failed to demonstrate any particularly inhibitory effects on MCF-7 cells, it did imply that a fraction of the SbE exhibited chemopreventive properties (94). Researchers found that berberine inhibited the expression of VEGF and fibronectin in breast cancer cells that were induced by TPA, and it also inhibited the synthesis of fibronectin that was driven by VEGF. Placidinositol 3-kinase/AKT signalling pathway suppression blocks these effects. To treat breast cancer with antiangiogenesis, berberine inhibits the primary angiogenic factors, which could lead to an excitingly low toxicity and relatively active antitumour drug (95). Berberine successfully reversed resistance of HER2+ breast cancer cells to lapatinib by disruption of redox homeostasis. These effects of LAP‐R cells’ apoptosis by inducing ROS with berberine is critically enhanced in the presence of inhibition signal c‐MYC/pro‐NRF2 and stability of NRF2 activated at phosphorylating glycogen synthase kinase 3β (96). Together, they provide evidence for the utility of berberine as an adjuvant therapy in addressing a range of resistance and survival pathways in breast cancer.

#### 2.3.2 Black cohosh

Black cohosh (*Actaea racemosa*) uses in managing menopausal symptoms has also been highlighted for breast cancer relevant to its possible symptomatic improvement of vasomotor symptoms such as hot flushes, night sweats, and mood swings reported in women undergoing tamoxifen or amrastase inhibitor therapy where oestrogen depleting would increase menopausal complaints (97). In contrast to phytoestrogens such as soy isoflavones, black cohosh does not seem to produce estrogenic type stimulation of breast or uterine tissues and its effects may be mediated by serotonergic and dopaminergic pathways, antioxidant sources, or anti-inflammatory actions (98). A number of randomized controlled trials and meta-analyses have described how standardized black cohosh extracts reduce hot flashes and sleep disturbance, while not increasing circulating oestrogen levels or accelerating tumour growth (99). Nevertheless, there are scant and unsystematic data about its direct anticancer effect, and generally the evidence is more solid for considering it as supportive care than as antitumour treatment. Rare hepatotoxicity, the heterogenicity of extract formulations available in the market, and loose regulatory control argue for liver function monitoring during long-term use as well as intake of clinically-trialled products (100).

#### 2.3.3 Green tea

Green tea (*Camellia sinensis*) and especially its bioactive catechin epigallocatechin-3-gallate (EGCG), is another widely investigated herbal extract with strong evidence for breast cancer prevention and adjuvant treatment (101). EGCG has anticancer effects such as the blockade of HER2 phosphorylation, regulation of angiogenesis and metastasis, phosphorylation and modulating of PI3K/Akt/MAPK signalling, and induction of apoptosis through mitochondrial pathways (102). In preclinical studies, green tea catechins have been found to increase the effectiveness of tamoxifen, trastuzumab and cisplatin; drinker’s levels of oxidant DNA damage also have decreased and antioxidant-enzyme activities in breast tissue improved. Asian populations showing a high green tea consumption have consistently observed reduced incidence or risk of breast cancer and lower rate of recurrences in regular tea intake, even though these associations that are shaped by genetic or lifestyle behaviours (103). Green tea extracts in breast cancer patients have shown evidences of promising but heterogeneous results, with some studies showing reductions in circulating levels of oestradiol and peroxidase tumour markers, and decreased risk of recurrence while other trials describes little or no clinical benefit due mainly to differences on dosage, duration and catechin bioavailability (104). Crucially, hepatotoxicity, gastrointestinal upset and potential warfarin- and chemotherapeutic-drug interactions have been reported in rare cases of dosing concentrated green tea at pharmacological levels.

*In vitro* and *in vivo* studies with MCF-7 breast cancer cells shown that EGCG had a strong anticancer impact. The involvement of miR-25 in the regulation of apoptotic marker genes via downregulation followed by recovery of response upon reconstitution, as well as the EGCG-mediated induction of apoptosis and interference with cell cycle progression (G2/M block), provide evidence that miR-25 may be a regulator of the apoptotic effects created by EGCG in these cells. By lowering Ki-67 and increasing poly(ADP-ribose) polymerase 1, EGCG increased apoptotic signalling and prevented tumour growth *in vivo* (105). Research has demonstrated that curcumin and EGCG, when administered together, inhibited breast cancer stem cell characteristics such as tumour sphere formation and CD44+ cell proliferation (23). In terms of the underlying process, it was observed that the combination of curcumin and EGCG inhibited the phosphorylation of STAT3 and the interaction between STAT3 and NF-κB, two crucial molecules for the survival of cancer stem cells. Using a topical EGCG solution as a prophylactic measure reduced the occurrence and extent of radiation dermatitis in breast cancer patient undergoing adjuvant radiotherapy, according to a randomised clinical trial (106). Utilising next-generation sequencing, almost 1500 miRNAs, both known and unknown, were discovered. EGCG controlled the expression of 873 known miRNAs and 47 unknown miRNAs. These microRNAs were associated with carcinogenic pathways in the breast cancer cell line MDA-MB-231, according to the bioinformatics study (KEGG and PANTHER) (107). All things considered, these findings point to the possibility that EGCG could function as a cancer preventative, therapeutic, and supportive agent in breast cancer, leading to the need for additional research and clinical testing.

#### 2.3.4 Oldenlandia diffusa (Willd.)

A well-defined traditional Chinese medicine made from the widely-spread plant *Oldenlandia diffusa* (Willd.) Roxb (108). Significant anticancer and antineoplastic properties were also revealed by *Oldenlandia diffusa* in pharmacological research (109). Notably, ODE stops cells from proliferating and urges them to die by activating p53 through ERα/SP1. Oleanolic acid and ursolic acid, two active chemicals in ODE, were also found to be capable of this, as we had suspected (110). *Oldenlandia diffusa* has anti-metastasis properties, including the ability to inhibit MCF-7 cell invasion through ERK, p38, and NF-kB signalling, as well as MMP9 and ICAM1 suppression and apoptosis regulation (111). Ursolic acid, isolated by bioactivity-guided fractionation of *Oldenlandia diffusa*, suppressed glycolytic metabolism and hence prevented the spread of breast cancer. This anti-metastatic effect was due to the activation of the SP1/caveoli-1 signalling pathway (112, 113).

Finally, *Oldenlandia diffusa* (Willd.) Roxb. is a common herb in Traditional Chinese medicine that shows great potential in the fight against inflammation and tumours. It has been shown that the *Oldenlandia diffusa* extract can regulate numerous signalling pathways, leading to apoptosis and growth suppression in breast cancer cells (114, 115). The bioactive chemicals responsible for this effect are ursolic acid and oleanolic acid. In addition, research has shown that *Oldenlandia diffusa* can limit the spread of breast cancer by influencing glycolytic metabolism and the molecular pathways that are essential for their anti-metastatic effects (116). This suggests that *Oldenlandia diffusa* could be an intriguing target for future therapeutic interventions.

### 2.4 Antioxidants and polyphenols

Antioxidants and polyphenols represent a diverse group of bioactive compounds with potential relevance in breast cancer management (117–119). They exert pleiotropic effects, including modulation of oxidative stress, regulation of inflammatory pathways, and interference with oncogenic signalling cascades. Preclinical studies consistently demonstrate their ability to inhibit tumour proliferation, induce apoptosis, and enhance sensitivity to conventional therapies (120–122). However, while these mechanistic insights are promising, the translation into robust clinical outcomes remains limited, and current evidence is largely derived from *in vitro* and animal models (123–125). Therefore, their role should be framed as supportive and investigational, pending validation through well−designed clinical trials.

#### 2.4.1 Flavonoids

Natural goods contain flavonoids, a class of chemicals with many bioactivities such as antibacterial, antioxidant, antimalarial, & antiallergic/anti-inflammatory properties (124, 126, 127). These substances help prevent heart disease, osteoporosis, breast cancer, and prostate cancer, much like phytoestrogens. Flavonoids and isoflavones have minimal inhibitory effects on aromatase and 17β-hydroxysteroid dehydrogenases (17β-HSD) in breast cancer cells, and they also hinder the generation of active oestrogens & steroid synthesis within cancer cells (128). The chemical structures of flavonoids classify them into several subgroups, including flavones, flavanones, flavanols, isoflavones, the leucoanthocyanidins, anthocyanins, and chalcones (129).

#### 2.4.2 Puerarin

The isoflavone puerarin, derived from the dried root of kudzu (*Radix puerariae*), has a long history of use as a medicinal ingredient in traditional Chinese medicine (130). Researchers relied on this traditional medicine for a long time to aid with inflammation (131), arrhythmias, and, most notably, primary hypertension, whereby it effectively lowers both blood pressure and heart rate. By expanding the intracranial artery, puerarin protects the brain from cerebral ischaemia and increases blood flow to the brain. Additional rat central spinal nerve root functions that it can disrupt include tetrodotoxin-resistant Na+ activity (132). There is substantial proof that puerarin can inhibit tumour growth. It hinders the growth of lymphoma, lung, liver, gastric, and colorectal cancers by damaging mitochondria, stopping the cell cycle, and inducing apoptosis (133–136).

Research has shown that puerarin can inhibit the division of breast cancer cells by reducing the cell death rate and increasing the synthesis of anti-apoptotic proteins via the ERα signalling pathway, in contrast to pro-apoptotic proteins (137). Liu et al. reported that puerarin alters gene expression, primarily via reducing the lipopolysaccharide (LPS) impact in MCF-7 cells through suppressing CCR7 and CXCR4 protein levels (138). The *in vitro* adhesion of LPS-treated MCF-7 and MDA-MB-231 cells to the extracellular matrix (ECM) was significantly reduced, and the cell-adherence components ICAM and VCAM were downregulated. As a result, puerarin can inhibit LPS-induced breast cancer cell migration, invasion, and adhesion (138). When used locally with chemotherapeutic medications like oxaliplatin for breast cancer treatment, puerarin improved chemotherapy sensitivity, suppressed oxaliplatin-induced EMT, and inhibited the CAXII enzyme, which prevented type III drug resistance and breast cancer metastasis (139).

#### 2.4.3 Isoliquiritigenin

The flavanone derivative isoliquiritigenin (ISL), a chalcone-based chemical derived from *Glycyrrhizae rhizoma*. It is found in liquorice and a few other plants. By increasing apoptosis and suppressing proliferation and angiogenesis, isoliquiritigenin can play the antitumour role in terms of its function. Isoliquiritigenin inhibits the PI3K/AKT pathway and the AA metabolic network, it may suppress cells from MCF-7 and MDA-MB-231, for instance (140); through the processes of cytochrome C release, cysteine protease activation, and membrane potential of mitochondria loss induces cell death. The unregulated growth of breast cancer cells is essential for carcinogenesis. Isoloquiritigenin led to apoptosis and autophagy-mediated cell death by decreasing cyclin D1 expression as increasing the number of cells in the sub-G1 phase, which inhibited the proliferation of breast cancer cells (141). Isoliquiritigenin suppressed COX-2, CYP4A, PGE p-PI3K, p-PDK, and p-AKT, which led to its apoptotic and migration-reducing actions on MDA-MB-231 and BT-549 cells.

The anti-cancer efficacy of isoliquiritigenin has attracted the interest of researchers studying the mechanisms including self-phagy, tumour cell differentiation, and the inhibition of new blood vessel creation. When tumours develop, angiogenesis is an essential process (142). New blood vessel creation keeps tumours alive, which reduces their ability to metastasise. One recent advance in cancer treatment is the ability to inhibit tumour angiogenesis, which in turn prevents the formation of metastatic sources and channels. There is a possibility that isoliquiritigenin can slow tumour angiogenesis and speed up cell death by blocking the VEGF/VEGFR-2 signalling pathway (143). The anticancer mechanism of isoliquiritigenin is based on its capacity to stimulate cell differentiation. It shows that isoliquiritigenin can cause breast cancer cells to undergo autophagy and apoptosis through mTOR. They also found that it takes at least 48 hours after induction to observe a possible elimination of breast cancer cells (144). When considered as a whole, iso-liquiritigenin exhibited activity against cancer by way of its many targets and signalling pathways. Particularly promising as possibilities for the treatment of breast cancer, its compounds offer more potent bioactivity (**Table 2**).

**Table 2:** List of reported phytochemicals with anticancer activity.

Phytochemical	Source	Tumour inhibition	Clinical relevance	References
Curcumin	*Curcuma longa* (Turmeric)	NF-κB/STAT3 inhibition, apoptosis induction, HER2/ER modulation	Reduces chemo side effects; poor bioavailability addressed via nanoparticles	(145)
Withaferin A	*Withania somnifera* (Ashwagandha)	EMT reversal, ROS generation, mitochondrial disruption	Synergizes with TRAIL and celecoxib; selective cytotoxicity	(146)
Luteolin	*Reseda luteola*, vegetables	PI3K/AKT/mTOR blockade, VEGF downregulation, apoptosis	Enhances tamoxifen sensitivity; anti-angiogenic effects	(57)
Resveratrol	Grapes, berries, red wine	YAP pathway inhibition, NRF2 activation, fibroblast modulation	Suppresses TNBC proliferation; improves microenvironment	(147)
Quercetin	Onions, apples, kale	EGFR inhibition, EMT marker suppression, miR-146a upregulation	Nanoparticle delivery improves efficacy and safety	(148)
Genistein	Soy isoflavones	Estrogen receptor modulation, apoptosis, angiogenesis inhibition	Used in hormone-sensitive breast cancer adjunctively	(149)
Epigallocatechin gallate	Green tea	DNA methylation modulation, oxidative stress reduction	Shown to reduce recurrence risk; enhances chemo response	(150)
Silymarin	Milk thistle	Cell cycle arrest, antioxidant activity, apoptosis via caspase activation	Used for liver protection during chemotherapy	(151)
Ginsenoside Rg3	*Panax ginseng*	VEGF suppression, caspase activation, EMT inhibition	Reduces fatigue; improves survival in East Asian cohorts	(152)
Berberine	*Berberis* species	AMPK activation, mitochondrial apoptosis, telomerase inhibition	Promising in TNBC models; limited clinical trials	(153)
Thymoquinone	Black seed (*Nigella sativa*)	p53 activation, ROS-mediated apoptosis, anti-inflammatory	Shown to reduce tumour volume in animal models	(154)
Astragalus polysaccharide	*Astragalus membranaceus*	Immune modulation, apoptosis, angiogenesis inhibition	Used in TCM; improves chemo tolerance	(155)
Boswellic acid	*Boswellia serrata* (Frankincense)	5-LOX inhibition, apoptosis induction, anti-inflammatory	Reduces inflammation; under clinical investigation	(156)
Honokiol	Magnolia bark	ERK inhibition, mitochondrial apoptosis, angiogenesis blockade	Enhances chemo efficacy; neuroprotective adjunct	(157)
Indole-3-carbinol	Cruciferous vegetables	Estrogen metabolism modulation, DNA repair enhancement	Used in hormone-sensitive cancers; dietary relevance	(158)

#### 2.4.4 Apigenin

Among the many plant foods and plants that contain the flavonoid apigenin are onions, grapefruit, and parsley, among many others. Dou et al. used a 70% methanol solution to isolate apigenin from *G. veitchiorum* flowers; HPLC-MS/MS was used to identify its components (159). One possible mechanism for the antineoplastic effect is the inhibition of IL-6 signalling pathways, which suppresses cellular invasion, and another is the abrogation of cell cycle progression in breast cancer cells (specifically MDA-MB-231 cells) via the down-regulation of important cyclins and CDK1 (160). Apigenin prevented the generation of cytokines linked to breast cancer carcinogenesis, such as CCL2 generated by TNFα and cytokines recruited by LPS. Apigenin upregulates p53 and initiates the Caspase cascade in MCF-7 cells, which leads to ROS generation, apoptosis, and G2/M phase arrest; the effects of this cascade are dose-dependent (161).

**Figure 2:**
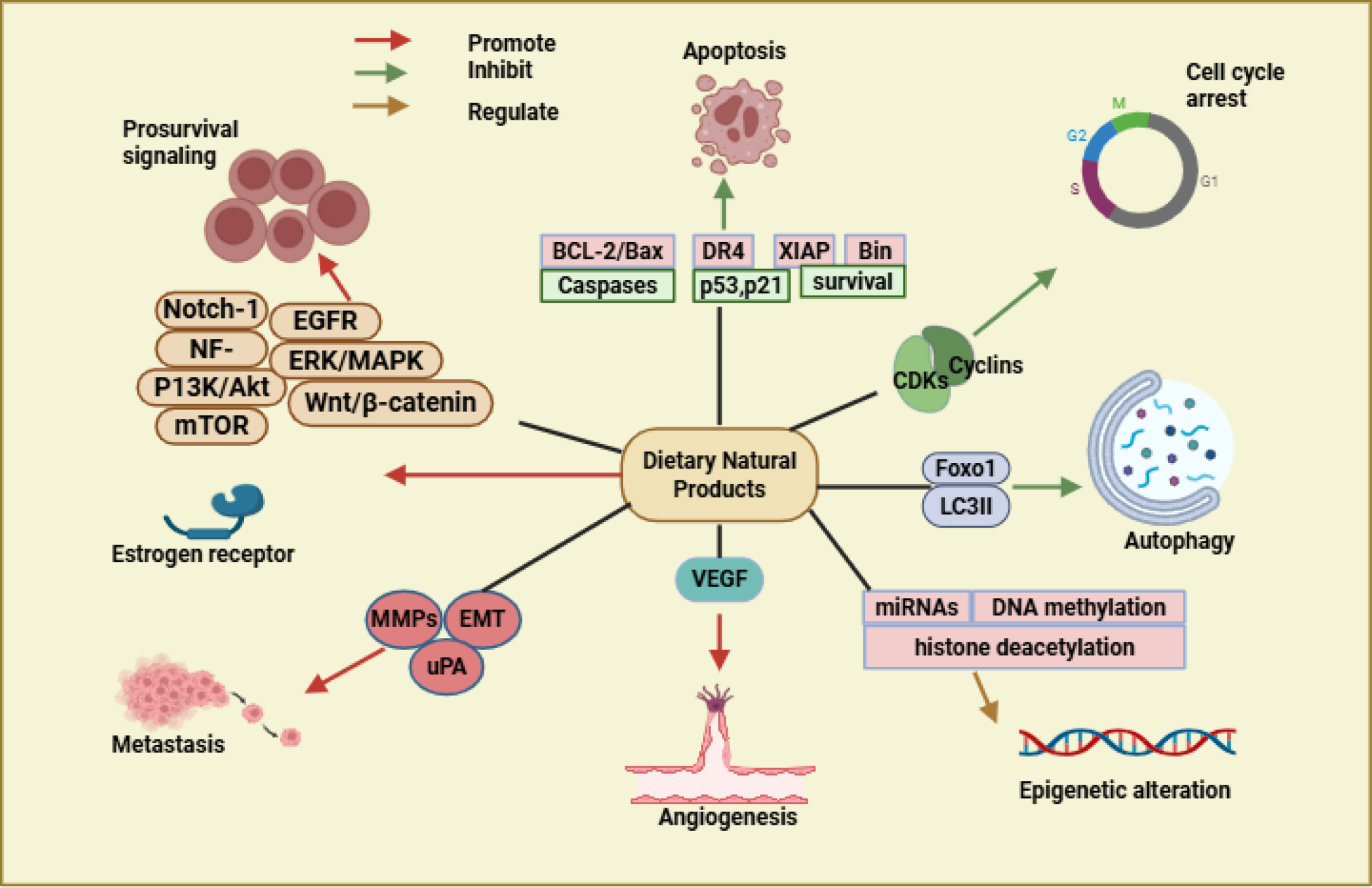
Mechanistic effects of dietary natural products on relevant signalling pathways in breast cancer biology. Created in BioRender. https://app.biorender.com/illustrations/68ef4761a2eb4bab5ee65f2d. Several dietary bioactives (polyphenol, flavonoid, alkaloid and terpenoid) are shown in the figure affecting various pathways associated with cancer. The compounds induce apoptosis by activating caspases, p53, p21, and death receptors (DR4) and at the same time inhibit the anti-apoptotic proteins, BCL-2, XIAP, and Survivin. Across G1, S, G2, and M phases, a cell cycle arrest is ultimately induced via the downregulation of multiple cyclins and CDKs. Foxo1 and LC3II signalling in regulating autophagy. Epigenetic modifications consist of miRNA, DNA methylation, and histone deacetylation. VEGF inhibition prevents angiogenesis. Proven to decrease the metastatic potential, by targeting matrix metalloproteinases (MMPs), epithelial–mesenchymal transition (EMT) markers and urokinase-type plasminogen activator (uPA) Downregulation of pro-survival signalling pathways (Notch-1, NF-κB, PI3K/Akt, mTOR, EGFR, ERK/MAPK, and Wnt/β-catenin). This also impacts signalling through the estrogen receptor, changin its relevance to certain subtypes of hormone-responsive breast cancer.

By combining apigenin with p53 inhibitors, the apoptotic rate in MCF-7 cells was considerably decreased. This study also found that anti-doxorubicin produced inflammation and oxidative stress in the kidneys, and that doxorubicin plus apigenin increased resistance to doxorubicin in breast cancer cells via the JAK/STAT signalling pathway (162). Apigenin inhibits the growth of ER-positive breast cancer cells via its action as an oestrogen receptor antagonist and its potential regulation of the AKT/FOXM1 pathway (163). Apigenin may be able to reverse resistance to endocrine therapy therapies. An aggressive and drug-resistant form of breast cancer, known as FOXM1 overexpression, is associated with a dismal prognosis. Apigenin increased production of cleaved caspase-3, cleaved caspase-8, and cleaved PARP, which inhibited the proliferation and colony formation of HER2+ BT-474 cells (160). Apigenin also reduced the growth of HER2+ BT-474 cell by reducing the transcriptional activity of VEGF and STAT3. The expression of phosphorylated STAT3, JAK1, and JAK2 was also reduced (164), which reduced VEGF secretion and nuclear phosphorylated STAT3 translocation for CoCl2-stimulated HER2+ BT-474 cells (**Figure 2**).

### 2.5 Omega-3 fatty acids and dietary oils

Some of the most studied nutritional supplements for breast cancer are omega-3 fatty acids and dietary oils due to their antiproliferative, immunomodulatory, or anti-inflammatory properties (165). These properties align with the primary pathways involved in tumourigenesis, progression, and treatment response. Marine oils such as fish oil and algae oil are rich in omega-3 polyunsaturated fatty acids (PUFAs) like eicosapentaenoic acid (EPA) and docosahexaenoic acid (DHA). These PUFAs are different from omega-6 PUFAs like arachidonic acid and play important roles in tumour microenvironment through competitive integration into cell membranes and in enzymatic metabolism through the cyclooxygenase-2 (COX-2) or lipoxygenase pathways.

Breast cancer risk factors include chronic inflammation, which can be mitigated by decreasing the body’s synthesis of eicosanoid molecules that promote inflammation, such as prostaglandin E2 (PGE2) and leukotrienes. Resolvins and protectins, which are anti-inflammatory mediators, are subsequently produced because of this event (166). Experimental studies have shown that EPA and DHA can inhibit the growth of breast cancer cells by interacting with oncogenic signalling pathways like PI3K/Akt, MAPK/ERK, NF-κB, & Wnt/β-catenin. They can reduce levels of Bcl-2 and increase levels of Bax and caspase-3, two proteins that promote cell death, and trigger mitochondrial mediated apoptosis. Further, they can decrease the expression of the VEGF, which suppresses angiogenesis, and they can inhibit matrix metalloproteinases (MMP-2, MMP-9) which inhibit the potential for metastasis (167). It is interesting to note that when combined with conventional chemotherapy drugs such as doxorubicin, cyclophosphamide, and taxanes, these fatty acids improve cancer cells’ sensitivity to cytotoxic drugs, lessen side effects like cachexia, neuropathy, or myelosuppression, or overcome multidrug resistance (168).

Research into the possible adjuvant effects of omega-3 fatty acids derived from non-fish sources in breast cancer has also been conducted. Reducing oxidative DNA damage, circulating oestradiol levels, and estrogen-dependent breast cancer growth were all shown in preclinical research (169). Whether flaxseed oil possesses phytoestrogenic or antioxidant properties is an open question. The oil contains lignans and alpha-linolenic acid (ALA). Women who have just received a breast cancer diagnosis have shown conflicting results from clinical trials that tested flaxseed’s impact on tumour proliferation markers (i.e., Ki-67) (170). Research has shown that consuming olive oil-a staple of the Mediterranean diet—may lower the risk of breast cancer and its complications. This is due to its many health benefits, including its anti-inflammatory, antioxidant, and possibly modulating effects on oestrogen hormone receptor signalling or the stimulation of DNA repair pathways. Additionally, olive oil is rich in monounsaturated fatty acids (MUFAs) or phenolic compounds like hydroxytyrosol (171).

Translationally, incorporation of omega-3 fatty acids and dietary oils into the context of breast cancer treatment require a precision nutrition approach based on natural production vs mimicking chemical derivatives for clinical practice, supplements with standardized formulas containing optimal ratios of EPA/DHA, advanced delivery methods such as nanoemulsions that increase bioavailability, multicentre randomized controlled trial studies (ideally large-scale) focusing thematically on meaningful endpoints related to clinical outcomes including OS and RFS (172). Interaction between omega-3 PUFAs and tumour molecular subtypes (e.g., ER+, HER2+, TNBC) and from host genetic variation in fatty acid metabolism (e.g., FADS1/FADS2 polymorphisms); it should be further investigated, specifically to identify patient populations most likely to benefit from supplementation (173). Also, the ability of omega-3 fatty acids to enhance or interfere with the effectiveness and toxicities of ICIs and other targeted agents is clinically novel in integrative oncology.

### 2.6 Probiotics and gut microbiota modulation

The human gut microbiome has rapidly emerged as a critical and modifiable cancer susceptibility, treatment-response, and survivorship determinant, including in breast cancer where the paradigm has attracted attention to probiotics and gut-microbiota targeting as promising adjunctive strategies (174). Mechanistically, an expanding body of preclinical/translational research suggests that the gut-microbiome impacts breast carcinogenesis and therapy through several interrelated pathways-chiefly in terms of its estrobolome (microbial pool of genes dedicated to estrogen metabolism), bioactive microbial metabolite synthesis, systemic/mucosal immunity modulation (influencing T-cell subsets/myeloid cell function/inflammatory cytokine networks) also together with drug pharmacokinetics/pharmacodynamics (175). These mechanisms collectively suggest that modifying population-wide or individual-level demographics in the microflora through live microorganisms/probiotics, fermentable substrates/prebiotics/synbiotics, combination derivations/postbiotics/dietary exposures/faecal microbiota transplantation (FMT) might exert influence on overall BC risk/treatment sensibilization-tolerability/quality-of life improvement.

Clinically, observational studies have linked specific microbiome unit reduced microbial diversity, depletion of butyrate-producing taxa, and an altered estrobolome to higher breast cancer risk or more aggressive phenotypes, and small interventional trials and pilot studies have explored probiotic supplementation (commonly Lactobacillus and Bifidobacterium strains) (176), showing encouraging signals such as improvement in systemic inflammatory markers, attenuation of gastrointestinal and mucosal toxicities during chemotherapy, mitigation of antibiotic-associated diarrhoea, modest improvements in metabolic parameters and bone health, and favourable changes in gut microbial composition; moreover, in the context of immunotherapy and targeted agents there is compelling translational evidence primarily from murine models and observational cohorts in other cancers that specific taxa enhance antitumour immune responses and predict checkpoint inhibitor efficacy, suggesting analogous potential in subsets of breast cancer patients, particularly those receiving immunomodulatory regimens (177).

## 3 Mechanisms of action of natural supplements against breast cancer

### 3.1 Anti-inflammatory and antioxidant pathways

The chronic inflammatory microenvironment in the breast activates nuclear factor-κB (NF-κB), cyclooxygenase-2 (COX-2), signalling transducers and activators of transcription-3 (STAT3), activator protein-1, and mitogen-activated protein kinase signalling cascades. This results in an overproduction of pro-inflammatory cytokines such as interleukin 6 (IL-6), tumour necrosis factor α, adhesion molecules, prostaglandins, and mitogen-activated protein kinase signalling cascades. The recruitment of TAMS, myeloid derived suppressor cells (MDSCs), regulatory T-cells, and regulatory T-cells all of which inhibit antitumour immunity contributes to this proneoplastic environment. At the same time, oxidative stress, which is caused by mitochondrial damage, activation of NADPH oxidase, and other environmental or treatment-related insults, promotes tumourigenesis through a cascade of events including: oxidative DNA adducts (8-oxo-dG), lipid peroxidation (4-HNE), protein oxidation, redox-regulated oncogene activation and mutagenesis, inhibition of the DNA repair machinery, and a vicious cycle of genomic instability accompanied by inflammatory signalling (178–180).

According to reports, these natural compounds have both direct and indirect antioxidant effects. Direct antioxidants scavenge free radicals, chelate metal ions, and inhibit lipid peroxidation. Indirect antioxidants restore cellular redox balance and safeguard biomolecules from oxidative stress by activating the Nrf2-Keap1/ARE pathways and inducing the expression of glutathione synthesis, superoxide dismutase (SOD), catalase, and GPx or peroxiredoxins (181). In addition to their anti-inflammatory effects, these compounds have inhibitory effects on the following pathways: inflammasome (NLRP3), Janus kinase/signal transducer and activator of transcription (JAK/STAT) pathway, endothelial cell vascular cell adhesion molecule-1 (VCAM-1) or intercellular adhesion molecule-1 (ICAM-1), matrix metalloproteinases involved in remodelling and metastasis (MMP 2, MMP 9), and chronic cycloxogenase derived prostaglandins that promote angiogenesis and immunosuppression (182). These overlapping effects not only inhibit tumour initiation and early progression but also render conventional therapies more efficacious – sensitizing tumour cells to chemotherapy-mediated apoptosis, protecting normal tissues against radiation-induced oxidative damage, dampening chemotherapy-associated inflammation (e.g. mucositis, neuropathy) and lowering systemic inflammatory markers (CRP, IL-6/TNF-α) associated with poor prognosis (183).

### 3.2 Hormonal modulation and estrogen receptor interactions

Hormonal modulation and ER interactions is perhaps the most extensively investigated mechanistic axis through which natural supplement act on breast cancer biology considering estrogen signalling being the main agent for carcinogenesis, progression and therapeutic resistance especially in HR positive subtypes that comprise almost 70% of total breast cancer patients worldwide (184). The estrogen-ER pathway is central to breast carcinogenesis, circulating oestrogens e.g. oestradiol (E2) bind ERα or ERβ in mammary epithelial cells, followed by receptor dimerisation, nuclear translocation and direct transcriptional regulation of EREs in promoter regions on target genes that regulate cell proliferation – cyclin D1, c-Myc -, survival – Bcl-2 or survivin -, angiogenesis – VEGF -, EMT – Snail, Twist -, metastasis MMPs (185), as non-genomic oestrogen signalling via membrane-localized ER and G protein–coupled oestrogen receptor (GPER) leads to rapid stimulation of PI3K/Akt, MAPK/ERK and Src pathways interacting further impetus to mitogenic and anti-apoptotic effects. Additionally, aromatase (CYP19A1) is expressed by breast adipose stromal cells and cancer-associated fibroblasts, which plays a crucial role in oestrogen production both locally and systemically, particularly in women who undergo through menopause (186). The resistance to endocrine treatments including tamoxifen, aromatase inhibitors, and selective ER degraders is protected by dysregulation of these pathways, which in turn fuel hormone-dependent tumour growth (187, 188).

Genistein and daidzein can downregulate ERα expression, to decrease cyclin D1 transcription and aromatase activity leading to diminish estrogen biosynthesis suggesting a modulation of CM-mediated TME-oestrogen-signalling. Lignans from flaxseed, converted to enterolactone (EL) and enterodiol by gut microflora within the intestines, possess weak estrogenic activity that competes with endogenous oestrogens for ER binding causing decreased mitogenic stimulation and lower circulating estrogen concentrations via upregulation of sex hormone–binding globulin (SHBG) (189, 190).

Indole-3-carbinol (I3C) and its metabolite DIM exert hormone-modulatory effects by shifting estrogen metabolism from the genotoxic 16α-hydroxyestrone pathway toward the less carcinogenic 2-hydroxyestrone pathway, enhancing CYP1A1/1A2 activity, and simultaneously downregulating ERα and upregulating ERβ expression, which together lead to suppression of estrogen-driven proliferation and sensitization of tumours to endocrine therapies (191). Resveratrol, a polyphenolic stilbene, displays concentration-dependent biphasic actions, at low doses it can weakly mimic estrogenic effects via ERβ activation, whereas at higher pharmacologic concentrations it acts as an ER antagonist, inhibits aromatase transcription, and disrupts co-activator recruitment to the ERα complex, thereby exerting anti-proliferative and pro-apoptotic effects in ER-positive breast cancer cells (192).

### 3.3 Immune system enhancement

The natural substances can act as immune checkpoint and co-inhibitory modulators, some polyphenols decrease the expression of tissue-and tumoural PD-L1 or of CTLA-4 on T cells giving a mechanistic reasoning to combination with nutritional support in patients treated with check point inhibitors, eventually reversing acquired resistance to treatment or decreasing the effective dose for drug administration (193). Many small randomized trials and observational studies have shown increases in NK cell cytotoxicity, improvements in vaccine response, decreases in infection rates (via antiviral or stimulatory therapy), declines in chemotherapy-associated lymphopenia, and improved patient-reported outcomes (fatigue, QoL) with concomitant use of specific supplements (e.g., mushroom extracts/beta-glucans/ginseng); however, no large powered studies show significant survival or recurrence benefits secondary to immunomodulation (194). Safety and translational challenges are considerable and must drive clinical application: immune stimulation carries theoretical risk as well as known risk of worsening autoimmune phenomena or inflammatory toxicities, particularly in the setting of active immunosuppression (e.g., newly diagnosed with cytotoxic therapy, on high-dose steroids); live probiotics or incompletely characterized biologics extracts can result in infection/invasion or translocation; batch-to-batch variation, contamination with pathogens or lead/arsenic, product- product variability users assume is uniform (but can be diverse within a supply chain), herb-drug interactions like modulating CYP enzymes that change the pharmacokinetics of drugs with narrow therapeutic windows all contribute to problems related to reproducibility and safety (195).

**Figure 3:**
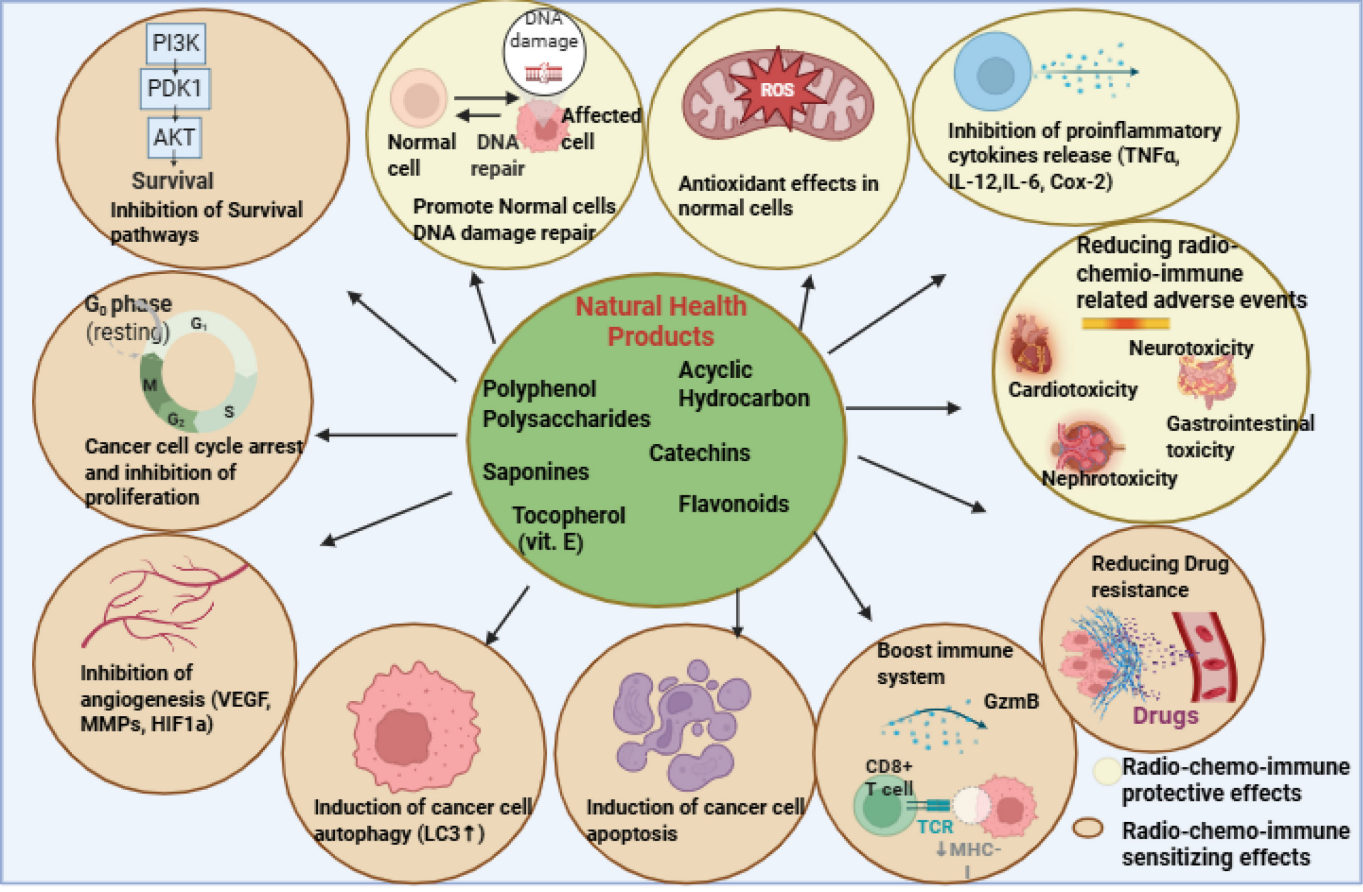
Diverse anticancer and immunomodulatory effects of natural health products. Created in BioRender. https://app.biorender.com/illustrations/68ef6845aca37e683af350b1. Overview of the varied cellular and immunological mechanisms of action whereby natural products exert anticancer effects and modify immune responses related to therapies. Figure showing various bioactive compounds that represents the potential therapeutic activity of polyphenols, polysaccharides, saponins, catechins, tocopherol (vitamin E), acyclic hydrocarbons, and flavonoids. The core is surrounded by eight mechanistic modules: inhibition of survival signalling (PI3K, PD1, AKT, mTOR), modulation of DNA damage and repair, antioxidant and anti-inflammatory actions (TNFα, IL-1β, IL-6, Cox-2), suppression of angiogenesis (VEGF, MMPs, HIF1α), blockade of metastasis, interference with autophagy (LC3↑), and regulation of apoptosis. The bottom part emphasizes their function in ameliorating radio-chemo-immune detrimental effects (neurotoxicity, cardiotoxicity, gastrointestinal and renal toxicity), increasing immune defence through CD8 + T cells and NK cells (granzyme B, perforin), and sensitizing tumour cells to therapy by reversing drug resistance.

Mechanistic clinical studies with integrated immunophenotyping and multi-omics linked to immune response biomarkers (NK activity assays, T-cell clonality, cytokine panels, tumour immune infiltrate changes) GMP-formulated products (defined active components/valid potency) (**Figure 3**). Development of next-generation strategies-(i.e. engineered probiotic strains for selective delivery of immuno-stimulatory molecules to tumours; nanoparticle formulations co-delivering immumodulators + checkpoint inhibitors; rational combinations that have mitigated effects while maximizing anti-tumour immunity (196). Ultimately, implementation of immune-augmenting nutritional supplements within breast cancer care may require personalized approaches taking into consideration tumour subtype, underlying immunologic competency/deficiency, previous treatments received, microbiome profile and comorbid autoimmunity as well as multidisciplinary teamwork to translate promising immunobiologic signals into safe and empirically defined adjuncts to conventional anticancer immunity that are additive rather than counterproductive to patient health (197).

### 3.4 Epigenetic and signal transduction effects

The regulation of epigenetics and the manipulation of signal transduction pathways have emerged as a fundamental mechanistic basis by which natural agents can exert strong anti-cancer effects in breast cancer, affecting the various stages of tumour development (initiation, progression, metastasis), and response to therapy by targeting diverse interactions between gene expression, chromatin structure, and intracellular signalling elements (198). At the core of breast carcinogenesis is epigenetic disruption, which involves changes in DNA methylation, histone modifications, and non-coding RNA. This disrupts tumour suppressor genes and activates oncogenes. Epigenetic disruption is also a component of hormonal resistance and the mechanisms of endowing hormones. Various signalling cascades, such as PI3K/Akt/mTOR, MAPK/ERK, Wnt/β-catenin, the NF-κB pathway, JAK/STAT pathway, and TGF-β cytokine signalling, collaborate to orchestrate the proliferation, survival, invasion, and metastatic proficiency of malignant cells. Green tea EGCG inhibits DNMTs leading to hypomethylation of the tumour suppressor gene promoters (e.g., p16, RARβ) and re-expression of pro-apoptotic genes as well as it interferes with RTK signalling NF-κB activation that are associated with inflammatory and proliferative signals (16). Resveratrol hinders the PI3K/Akt/mTOR & Wnt/β-catenin pathways, which result in cell cycle arrest and apoptosis, and activates SIRT1, a NAD+-dependent deacetylase, which impacts histone acetylation and p53 actions. Resveratrol also has dual effects on epigenetics and signalling. Cruciferous vegetables contain sulforaphane, the most powerful isothiocyanate. It inhibits histone deacetylase (HDAC) and modulates DNA methyltransferase (DNMT) activity. These effects could lead to suppressing cyclin D1, re-expressing silenced tumour suppressor genes, inhibiting inflammation mediated by NF-κB, or downregulating metastatic markers like MMP-2 and MMP-9. DIM and I3C enhance the effects of anti-estrogen treatments by acting on AhR-mediated transcription, changing the epigenetics of estrogen-responsive genes, and suppressing ERα signalling (199).

## 4 Safety challenges and drug-supplement interactions

The integration of natural supplements into breast cancer therapy requires careful consideration of pharmacokinetic and pharmacodynamic interactions. Several bioactive compounds modulate cytochrome P450 enzymes, particularly CYP3A4, CYP2D6, and CYP2C9, which are essential for the metabolism of chemotherapeutic agents and endocrine therapies. For example, interference with CYP2D6 activity may influence tamoxifen bioactivation, potentially affecting therapeutic efficacy. Additionally, antioxidant supplementation during chemotherapy raises concerns regarding attenuation of reactive oxygen species–mediated cytotoxic mechanisms. Interactions with immune checkpoint inhibitors and targeted therapies remain incompletely characterized, underscoring the need for pharmacovigilance and individualized risk assessment. These considerations highlight the importance of evaluating supplements within a clinically integrated framework rather than as isolated adjuncts.

### 4.1 Risks of supplement use during chemotherapy

The use of natural remedies during chemotherapy in breast cancer is a compound scenario that includes the safety issue as well as drug–supplement interferences and merits meticulous assessment, because these interventions—usually considered as harmless substances—may significantly alter the pharmacokinetic/pharmacodynamic response, antitumoural activity, and toxicity profile to conventional oncologic treatment (200). To reach their full anti-cancer potential, cytotoxic agents like doxorubicin, epirubicin, paclitaxel, docetaxel, cisplatin, carboplatin, antimetabolites, 5-fluorouracil, capecitabine, and targeted therapies like trastuzumab and CDK4/6 inhibitors rely on precise dosing or predictable metabolic pathways, among other factors (201). The metabolism of many chemotherapeutic and endocrine medications is facilitated by cytochrome P450 (CYP) enzymes such CYP3A4, CYP2D6, and CYP2C9, which provide a significant obstacle due to drug-drug interactions (202).

St. John’s Wort, ginkgo biloba, goldenseal, or high-dose green tea extracts are all potent CYP modulators. St. John’s wort induces CYP3A4 and P-glycoprotein (P-pg), which lowers the plasma concentration of taxanes or tamoxifen and may decrease efficacy. On the other hand, ginkgo and some flavonoids may inhibit CYP3A4 or Pg-pg, leading to increased drug exposure and an increased risk of toxicity, such as myelosuppression, neuropathy, or hepatotoxicity (203). Preclinical data suggest that antioxidant supplements may protect tumour cells from oxidative damage; however, clinical relevance is controversial because some studies reveal reduced chemotherapeutic efficacy when antioxidants are taken in combination with high doses of selenium and polyphenols. Antioxidant supplements, which include vitamins C and E, high-dose selenium, and polyphenols, may also interfere with chemotherapy’s ROS-mediated cytotoxicity (204).

Hematologic toxicities are another significant safety issue since many of the supplements have inherent immunomodulatory or anti-platelet activities. For example, omega-3 fatty acids ginkgo biloba garlic extracts high-dose vitamin A may increase bleeding risk via a reduction in platelet aggregation; this is of particular concern in patients receiving thrombocytopenia-related or co-administered chemotherapeutics (205). Furthermore, immunostimulatory adjuvants including β-glucans, medicinal mushrooms or high-dose probiotics can also in immuno-compromised patients induce opportunistic infections, sepsis or bacteremia especially if the integrity of gut barrier is compromised by chemotherapy-induced mucositis (206).

Another important issue is the gastrointestinal tolerability and even attacks, nausea, vomiting, diarrhoea, or mucositis during chemotherapy; most of the herbal extracts and concentrated polyphenols may aggravate them currently which makes it difficult to support care and adherence to medication periods (207). Liver (hepatic) and kidney safety considerations are equally important, since some supplements that undergo hepatic metabolism or renal excretion can accumulate or interact with chemotherapeutic drugs leading to hepatotoxicity (e.g., kava, high-dose green tea extract) or nephrotoxicity in patients who already suffer from impaired organ function or taking chemotherapy agents known to be nephrotoxic such as cisplatin (208, 209). Some natural anti-inflammatory agents (curcumin, resveratrol and quercetin) can act by modulating NF-κB, STAT3 or MAPK signalling that may sensitize to chemotherapeutic treatments leading to protection from apoptosis when used inappropriately (210). The precise timing, dosage, formulation and level of purity of supplements are thus relevant to the safety issue; non-standardized, active compound varying (in concentration) or contaminated with heavy metals or microorganisms in OTC products is frequently introduced into this equation leading again to increased unpredictability.

### 4.2 Hepatotoxicity, nephrotoxicity, and cardiovascular concerns

Hepatotoxicity, nephrotoxicity and cardiovascular issues are the key safety concerns related to natural supplements use in breast cancer patients especially when combined with conventional chemotherapy, targeted drugs, or supportive agents (211). The liver, which is the main organ for drug metabolism, is at a particular risk of adverse effects with respect to herbal and nutraceutical agents subject to extensive hepatic biotransformation or induction/inhibition of cytochrome P450 (CYP) enzymes (212). Dietary supplements, including green tea extract (particularly when standardized to have a high catechin concentration) and kava, black cohosh, high-dose curcumin, and some Chinese herbal preparations, are implicated in hepatocellular injury, cholestasis, or fulminant hepatic failure in both single case reports and small clinical series.

Potential of these effects may be heightened in patients with breast cancer treated with hepatically metabolized chemotherapeutics (e.g., cyclophosphamide, paclitaxel, anthracyclines) or tyrosine kinase inhibitors in which drug-drug interaction-based elevation or lowering of enzyme activity can result in supra-therapeutic drug concentrations, accumulation of toxic metabolites and compromised detoxification of reactive oxygen species facilitating hepatocellular distress. The risk of hepatotoxicity is also modulated by background liver disease, obesity or chemotherapy-related fatty liver (as in steatosis), alcohol consumption and genetic mutations in CYP2C9, CYP3A4 and UGT1A1 (213). Kidneys are another potential target of supplement-induced toxicity, owing to the facts that many herbal constituents and potent nutraceuticals undergo renal excretion or may modulate renal haemodynamics and tubular functions (214). Large amounts of ginseng, aristolochic acid-containing herbs and some green tea extracts cause acute tubular necrosis, interstitial nephritis, or CKD either alone or in combination with nephrotoxic chemotherapy agents (e.g., cisplatin, carboplatin; or methotrexate). Agents affecting fluid balance or electrolytes (e.g., liquorice root; high-dose vitamin D; omega-3 fatty acids) have the potential to exacerbate preexisting kidney insufficiency (215).

Cardiovascular safety considerations are also of utmost concern since cardiotoxicity is often an intrinsic risk associated with breast cancer treatments. Anthracyclines (as well as trastuzumab) are characterized by dose-dependent cardiomyopathy, arrhythmias, and left ventricular dysfunction (216), with some tyrosine kinase inhibitors associated to QT prolongation or increased blood pressure. These effects can be compounded by natural supplements through a variety of mechanisms, herbal stimulants, high-dose ephedra or green tea extracts, and some flavonoids increase heart rate, blood pressure, or sympathetic tone; omega-3 fatty acids and garlic may potentiate anticoagulation in patients on antiplatelet or anticoagulant therapy such that these patients are at risk for haemorrhage. The constituents of supplements with unknown variability can unpredictably interact with cardiac ion channels or CYP-mediated drug metabolism causing alterations in drug exposure and cardiotoxic potential (217). In addition, the pro-oxidant/antioxidant effects of polyphenols and vitamins are context-dependent and moderate doses may be cardioprotective while supraphysiologic levels negatively affect the oxidative state or disrupt ROS-mediated signalling that is important for chemotherapy-induced killing of tumour cells (218). **Table 3** showing summary of natural supplements in breast cancer.

**Table 3:** Summary of natural supplements/compounds in breast cancer: targets, evidence, and therapeutic potential.

Supplement/compound	Major target pathways	Evidence level	Clinical relevance/key findings
Curcumin	NF-κB, STAT3, COX-2, p53, Nrf2	Preclinical (strong) Clinical (moderate)	Induces apoptosis and antioxidant defence; 45% tumour reduction in mice; improved chemo response in small trials (500 mg BID, safe up to 4 g/day).
Resveratrol	ERα/β, PI3K/Akt/mTOR, SIRT1, AMPK	Preclinical (strong) Clinical (limited)	Acts as phytoestrogen; ↓ IGF-1 ~ 35% in Phase I trial (1 g/day); modulates metabolism and reduces oxidative stress.
Quercetin	HER2, EGFR, MAPK, Bcl-2/Bax, Aromatase	Preclinical (strong) Clinical (limited)	Cytotoxic & anti-proliferative; enhances doxorubicin efficacy 1.6×; modulates estrogen synthesis; safe ≤1 g/day orally.
Luteolin	Akt/mTOR, Caspase-8/9, MMP-2/9, GSH	Preclinical (moderate)	Anti-metastatic and antioxidant; ↓ tumour burden ~ 55% in mice; not yet validated clinically.
Withaferin A	NF-κB, JAK/STAT, Hsp90, Vimentin	Preclinical (strong)	Induces apoptosis and autophagy; ↓ tumour size 52%, ↓ NF-κB 75%; potent but limited toxicity data in humans.
Genistein/Soy Isoflavones	ERβ, PI3K/Akt, MAPK	Preclinical (strong) Clinical (moderate)	Selective estrogen modulation; improved QoL and ↓ hot flashes in breast-cancer survivors; safe dietary doses 50–100 mg/day.
EGCG (Green Tea Catechins)	VEGF, NF-κB, AMPK	Preclinical (strong) Clinical (emerging)	Anti-angiogenic; ↓ VEGF ~ 40% in cell models; modest biomarker effects in pilot human studies; safe ≤800 mg/day.
Omega-3 Fatty Acids (EPA/DHA)	NF-κB, COX-2, PPARγ	Clinical (strong)	Reduces inflammation and cachexia; ↓ IL-6 30%, improved QoL scores; safe up to 3 g/day; mild GI side effects.
Vitamin D3	VDR, PI3K/Akt, Wnt/β-catenin	Clinical (strong)	Associated with improved prognosis; serum 25(OH)D > 30 ng/mL linked to ↓ recurrence; safe 1,000–2,000 IU/day.
Probiotics (Lactobacillus spp.)	Gut–immune axis, NF-κB	Clinical (moderate)	↓ chemo-induced diarrhoea (–25% incidence); supports gut microbiota balance; well tolerated.

### 4.3 Translational Dose–Response Considerations and Human Attainability

While numerous *in vitro* studies report IC_50_ values demonstrating anti-proliferative or pro-apoptotic activity of natural compounds in breast cancer cell lines, the clinical relevance of these concentrations requires careful evaluation. Many reported IC_50_ values fall within micromolar ranges that may exceed achievable plasma concentrations following standard oral dosing in humans. Therefore, extrapolation from *in vitro* potency to clinical efficacy must be interpreted cautiously.

Pharmacokinetic studies of several bioactive compounds have demonstrated limited oral bioavailability, rapid metabolism, extensive first-pass hepatic clearance, and low systemic exposure. Peak plasma concentrations (Cmax) observed in human supplementation trials are often substantially lower than concentrations used in cell culture models. Furthermore, protein binding, tissue distribution, metabolic conjugation, and elimination kinetics further influence biologically active free drug levels.

In addition, tumour tissue exposure may not directly correlate with plasma concentration, and intracellular accumulation within malignant cells remains incompletely characterized for many compounds. These pharmacokinetic constraints highlight a translational gap between experimental conditions and clinically attainable dosing regimens.

Emerging strategies, including nanoformulation, liposomal encapsulation, and structural modification, aim to improve bioavailability and tissue delivery; however, robust clinical validation remains limited. Future translational studies should integrate pharmacokinetic–pharmacodynamic modelling to determine whether therapeutic concentrations observed *in vitro* are achievable and sustainable in human breast cancer patients.

Overall, while preclinical findings provide important mechanistic insights, clinical implementation must be guided by realistic exposure levels, safety margins, and well-designed human trials.

## 5 Translational perspectives

### 5.1 Preclinical models and biomarker development

The preclinical models and biomarker development represent the cornerstone of translational research towards incorporation of natural supplements in breast cancer therapy by adding mechanistic insights, validating efficacy, predicting therapeutic response, relevance towards patients for personalized therapy as well as on addressing their safety and dosing issues (219). *In vitro* studies of human breast cancer cell lines, including ER positive (MCF-7 and T47D), HER2+ (SK-BR-3) and triple negative (MDA-MB-231 and BT-549) models, have been very helpful in understanding the molecular mechanisms that regulate natural supplements as they modify growth progression, apoptosis, cell cycle control, invasion ability and metastatic capacity (220).

In addition to 2D cell culture, 3D organoid systems or co-culture systems with stromal fibroblasts, immune cells or endothelial cells have increased translational relevance by modelling the tumour microenvironment, heterotypic signalling and drug penetration kinetics (219, 220). For instance, organoid models have clearly shown that phytoestrogens and polyphenols can regulate ERα/ERβ signalling and aromatase activity within breast tissue-like architecture (221), whereas immune cell co-cultures have demonstrated that β-glucans, mushroom polysaccharides, as well as selected probiotics can increase NK cytotoxicity and DC maturation thereby providing a preclinical rationale to consider integrating immunomodulatory supplements into combinatorial therapy regimens (221).

*In vivo*, patient-derived xenografts (PDXs) and syngeneic mouse models have also demonstrated that CPEB3 depletion inhibits tumour growth and metastasis, as well as facilitating pharmacokinetics, bioavailability and lack of systemic toxicity evaluations prior to human testing which are invaluable safety and efficacy details (222). These models also enable the analysis of tumour-subtype specificity, interplay with chemotherapeutic or targeted compounds and potential organ-specific toxicities such as hepatic-, nephro- and cardiovascular-toxicity.

Biomarker research is paramount for translating preclinical results into precision medicine applications. Biological biomolecules based on gene expression, epigenetic alterations, microRNA profiles, protein phosphorylation and cytokine signatures can contribute to the selection of subsets of these patients for specific natural agents, monitoring *in vivo* biological efficacy and potential synergistic (or antagonistic) interactions with medical therapies (223). ERα/ERβ ratio and aromatase expression levels as well as 2-hydroxyestrone:16α-hydroxyestrone ratios are becoming used as biomarkers to direct phytoestrogen or DIM (diindolylmethane) supplementation, whereas the NF-κB, STAT3 and PI3K/Akt phosphorylation status potentially indicating curcumin, resveratrol or polyphenols use for chemosensitizing. Biomarkers of immune activation such as NK cell activity, CD8 + T cell infiltration, cytokine profiles (IL-12, IFN-γ and IL10), and Treg/MDSC ratios help identify populations of patients likely to benefit from immunomodulatory agents whereas circulating levels of polyphenols, isoflavones or glucosinolate metabolites serve as a marker for PK properties that can be utilized to optimize dosing and exposure (224).

### 5.2 Integrative oncology trials and protocols

The American Society of Clinical Oncology (ASCO) and the Society for Integrative Oncology have jointly formulated evidence-based approaches to the integration of complementary and alternative medicine (CAM) as applicable to the management of anxiety, depression, and fatigue and the use of cannabinoids and cannabis in the oncology setting. These guidelines are aimed to be evidence-based recommendations and they offer integrative modalities as adjuncts to conventional cancer treatment with the aim of improving clinical outcomes and quality of life (225).

Literature summarizes the integrative therapies, dietary and exercise interventions, mind-body therapies, acupuncture, yoga, massage, and dietary supplements for lifestyle modification purposes and symptom management in patients with breast cancer. Exercise holds significance, with a moderate 30 minutes of physical action daily, targeting 150 minutes a week. Meditation and yoga — which include the category of mind-body approaches — result in increased emotional self-regulation, anxiety, depression, and other Symptoms. It showed potential improvements for aromatase inhibitor-associated musculoskeletal symptoms, hot flashes, peripheral neuropathy and fatigue. Massage may be beneficial in treating pain, anxiety, stress, and QoL (226).

A systematic review included 45 randomized controlled trials (RCTs). The potential role of vitamin D supplementation appeared promising in a few studies when given alone or in synergy with other co-interventions, largely driving immunomodulatory and antioxidant effects of vitamin D. While beta-glucan and omega-3 fatty acids showed efficacy in reducing some symptoms and enhancing quality of life. Acetyl-L-carnitine and L-arginine are two amino acids for which some studies showed potential benefits; other studies have been less favourable. Beta-glucan had possible immune-enhancing effects, melatonin and creatine were of limited or no value for fatigue or muscle strength (227).

Integrative oncology studies and protocols are a key translational interface in which TS are being evaluated, with the aim to comprehensively evaluate their therapeutic potential, safety, pharmacokinetics and clinical efficacy, attempting to connect preclinical mechanistic findings with evidence-based clinical practice. Unlike other drug trials, integrative oncology studies encounter additional methodological issues such as the plethora of supplement formulations and their composition, variability in concentration of bioactive compounds across different source materials, patient-driven simultaneous use of products and multifaceted endpoints that include tumour response objective assessments as well as symptom control, quality-of-life evaluation, and biomarker modification (19).

Supplements like curcumin, green tea polyphenols, resveratrol, flaxseed lignans, omega-3 fatty acids, vitamin D, selenium, medicinal mushrooms extracts, probiotics, and soy isoflavones are being studied more as monotherapy adjuncts or in combination with chemotherapy and endocrine therapy for patients with postsurgical metastatic breast cancer. The studies are well-designed and rely on randomised controlled trials and observational studies (228). They are often multi-arm, dose-escalation trials designed to determine the appropriate therapeutic window in which optimal anti-tumour activity can be met, while reducing toxicity based on pharmacokinetics, bioavailability and patient compliance. For example, curcumin in nanoformulation and liposomal encapsulated has been investigated to circumvent poor systemic absorption and enhance targeting into tumour tissue to permit accurate discovery of dose-response relationship as well as pharmacodynamic effects *in vivo* (229).

Clinical protocols in integrative oncology studies commonly involve a detailed baseline assessment of tumour sub-type, receptor status, comorbidities, organ function, and concurrent medications including an organized evaluation of pre-existing and current use of supplements to adjust for possible confounding factors (230). Safety examinations are saved intensive and include serial hepatic, renal, cardiovascular function tests, haematology profiles and adverse event monitoring with special attention of combined administration of supplements with chemotherapeutics or endocrine agents rated to have organ-specific toxicity risk. Trials typically incorporate biomarker endpoints such as ERα/ERβ ratios, aromatase activity, plasma isoflavone or polyphenol concentrations, immune cell phenotyping, cytokine panels, oxidative stress markers and microbiome profile which are used to provide mechanistic read-outs of supplement activity and to inform patient stratification approaches (231). With immunomodulatory adjuvants, also NCCIs such as NK cell cytotoxicity, (TCD 8 T cells proliferations and do TIL density), PD-L1/CTLA-4 expression will provide the opportunity to correlate biologic activity with clinical response, for targeted use (precision medicine) (232).

New trial designs, such as adaptive, biomarker-driven, and crossover studies are increasingly used to overcome problems of inter-individual variability in response, bioavailability and metabolism (233). For instance, studies of phytoestrogens or lignans commonly include menopausal status, baseline levels of circulating estrogen and ability of gut microbiota to convert dietary precursors into bioactive metabolites in patient stratification leading to the determination of responding vs. non-responding individuals. Likewise, polyphenols or omega-3 fatty acid combinations with chemotherapy are studied for pharmacodynamic synergy by means of endpoints such as tumour volume reduction, progression-free survival and amelioration of chemotherapy-induced toxicity along with quality-of-life enhancement, keeping a close watch for antagonistic interactions or organ-specific adverse effects (234).

### 5.3 Patient-centred decision making

Patient-centred decision making is a key translation perspective in the integration of natural supplements to breast cancer therapy, simultaneously focusing on individualized patient care, informed consent, shared and rational decision making that aligns therapeutic options with patients values and preferences and potential of clinical context (235). Patient-centred strategies must identify patients’ tumour, current and planned conventional therapies (chemotherapy, endocrine therapy, targeted therapy and immunotherapy), comorbidities, organ function, nutrition profile, dietary habits and existing medication or supplement use since these factors critically impact both the efficacy and safety of supplementary approaches (236).

Patients are advised of the significance of standardized, GMP-certified product formulations, proper dosing and tailor it to timing relative to chemotherapy or endocrine therapy; other compliance monitoring is stressed for varied product quality and bioactive content can greatly affect safety and efficacy (237). Integrating biomarker-driven knowledge and translational research findings in counselling - like predictive responsiveness accorded to ERα/ERβ ratios, CYP enzymatic polymorphisms, gut microbiota or immune profiling—allows providing personalized recommendations as well as stratifying patients potentially responsive to specific interventions; hence establishing the link between preclinical mechanistic knowledge and clinical decision-making (238).

Patient-centred models similarly appreciate the significance of psychosocial and quality-of-life end points, such as fatigue, cognitive function, pain, emotional well-being and treatment-related nausea or neuropathy that are commonly endorsed by patients in explaining their motivation to take supplements (236). Integrative oncology protocols now include PROs, validated quality-of-life measures and symptom tracking tools to evaluate the global impact of natural supplements towards which adjustments in intervention can be iteratively made according to patient experience and tolerability. Good patient-centred decision making around the use of dietary supplements requires multidisciplinary care, involving oncologists, integrative medicine providers, clinical pharmacists, dietitians and nursing to monitor and mitigate risk as well as to fully document all supplement uses and be aware how they may interact with conventional therapy (239).

## 7 Clinical studies on breast cancer using natural supplements

In a study, subgroups of breast cancer were evaluated those without mammary abnormalities in the extra tumour epithelium glandular, those with non-proliferative breast cancer (n=127) and finally, 155 NPFCs and 185 PFCs were compared with their respective controls (n=903 vs 127 normal) (240). Using proliferative vs non-proliferative distortion of extra tumoural tissue, researchers evaluated the relative risk for breast cancer for NPFCs and PFCs. The palmitic and palmitoleic acid saturation index and the benign proliferative breast conditions—which may aid in breast cancer prevention—were negatively correlated with omega-3 fatty acid consumption. The risk was 67% lower when eicosapentaenoic acid (EPA) was inversely associated to NPFC. In another study, to find out soy isoflavones had any anti-cancer effects in breast cancer, this randomised, placebo-controlled trial performed for two years. For up to two years, 199 premenopausal women received part in the study; half received a placebo, and the other half took isoflavones. Researchers used either square root transformation or linear mixed-effects regression to look at the average changes in breast cancer (241). A biomarker for breast cancer risk was shown to be lowered in clinical trials including premenopausal women who took soy isoflavones. Since calcium was additionally marginally inversely linked with BMI and breast fat (241), soy consumption may provide novel health benefits for the prevention and treatment of breast cancer.

The effects of flaxseed on cancer of the breast patients who had passed through menopause were confirmed by researchers in a randomised, double-blind, placebo-controlled trial. 19 individuals were randomly assigned to receive either a 25 g flaxseed muffin or a placebo muffin for a duration of thirty-two days (242). Several tests were conducted on the tumour tissue upon patient diagnosis. These included ki-67 labelling index (main end point), c-erB2 expression, apoptosis, and oestrogen and progesterone receptors. Researchers also tracked the rate of lignan excretion during a 24-hour period. Apoptosis (30.7%), c-erbB2 expression (71.0%), and Ki-67 LI (34.2%) were all significantly reduced in the flaxseed group compared to the pre-treatment data (p = 0.001). The placebo group, on the other hand, showed no improvement. The possibility exists that the group given flaxseed also had an increased tendency to excrete lignan in their urine. Therefore, eating flaxseed may help breast cancer patients slow the growth of their tumours (170).

In a study of clinical trial Phase II of Polyphenol E, its effectiveness was assessed in forty women who were receiving adjuvant therapy for breast cancer stages I-III. Patients received either 400 mg of Polyphenon E (n=16), 600 mg (n=11), or 800 mg (n =3), or a placebo (n =10), per random assignment. Compared to the control group, those using Polyphenon E had reduced levels of the vascular endothelial growth factor (VEGF) from 2–4 months and significantly lower levels of serum hepatic growth factor (HGF) at 2 months. These findings raise the prospect that the therapeutic regulation of breast cancer by green tea polyphenols involves lipid metabolism, angiogenesis, and growth factor signalling (243). In a phase I study, eleven breast cancer patients were evaluated for their response to radiation therapy using doses of 3, 6, and 9 g of Trametes versicolour. Ultimately, the results proved that Trametes versicolour was both safe and beneficial in restoring bodily immunity. It is proposed that Trametes versicolour could have a reliable role in immunotherapy because higher doses are positively correlated with faster recovery (244).

In another investigation diagnosed with postmenopausal breast cancer participated in a phase 1 clinical study. They were given broccoli sprout extract (BSE) to take daily before surgery, with 200 µmol of isothiocyanate (ITC) being the active ingredient. Immunohistochemistry (IHC) allowed us to detect biomarkers in breast carcinoma tissue sections that were linked to immune checkpoint therapy (ITC) and chemo-resistant breast cancer. There was very little toxicity in the experiment; no individuals experienced toxic events of grade 4, and everyone adhered well to their prescribed therapy (245).

Researchers included 1442 breast cancer patients in our cohort analysis, with 710 serving as the test population and 732 as the control, in Switzerland and Germany. The experimental group underwent conventional therapy for a minimum of three months after receiving subcutaneous injections of *V. album* extract, while the control group only received standard treatment. Several symptoms disappeared more often in the systemic infection group compared to the one exposed to *V. album* extract, and survival rates were significantly longer. The results of the experiment confirm the idea that a STW-5 mixture with *V. album* extract can be safely administered as adjuvant therapy to patients via primary non-metastatic breast cancer. Furthermore, when taken in conjunction with conventional therapy, the occurrence of adverse drug reactions (ADRs) has been significantly reduced. Furthermore, compared to the control group, those given *V. album* extract had greater likelihood of surviving the trial (246).Modern studies have further substantiated the increasing role of integrative oncology involving the integration of mainstream cancer therapy and evidence-based dietary and lifestyle interventions to enhance patients’ prospects for recovery. For instance, in 2024, a randomized controlled trial in metastatic breast cancer evidenced the benefits of a whole-food, plant-based diet in terms of weight control, metabolic balance, and hormonal restorative effects among the patients undergoing systemic therapy. Some years were earlier, a 2024 meta-analysis demonstrated a 26% decrease in breast cancer recurrence related to soy isoflavone intake, primarily in the patient group of postmenopausal females with estrogen receptor–positive disease manifestations. In the meantime, a relatively recent review about the role of immunonutrition in oncology also illustrated the potential of omega-3 fatty acids, glutamine, and probiotics to achieve improved immune competence and reduced therapy-triggered inflammation. In addition to nutraceuticals, other integrative methodologies, such as lifestyle and dietary changes, have been substantiated in terms of enhanced quality of life, reduced fatigue scores, and prolonged survivorship among breast cancer patients.

## 6 Future directions

Research has been conducting due to the potential therapeutic and adjuvant benefits of bio-active dietary supplements for the therapy of breast cancer (247). Potential botanical medicines for the treatment of breast cancer include curcumin, resveratrol, green tea polyphenol, omega-3 fatty acids, calcium, vitamin D, and others with anti-proliferative, pro-apoptotic, anti-inflammatory, & anti-angiogenic properties. Translational research has started to find the molecular pathways affected by these supplements, which will help to close the gap between lab and clinical investigations. Among these pathways include NF-κB signalling, PI3K/Akt/mTOR branched modes of action associated to the oestrogen receptor, and epigenetic alterations (116). Notwithstanding about the promising mechanisms of action, translation to patients is limited by bioavailability and pharmacokinetic issues, inter-subject variability and possible interactions with other chemotherapeutic agents or endocrine therapies highlighting the need for well-conducted randomised controlled trials and PKPD studies (248).

It has also been focused on precision and personalization, with genomics, metabolomics, and microbiome profiles used to respond as early as possible to those patients likely to gain the most benefit from specific supplement interventions while limiting any unwanted side effects. Personalized supplementation might include optimization of dose, timing and natural agents combinations according to tumour molecular signature, patient immune status and lifestyle habits by integrating traditional oncology with precision nutraceutical medicine (249). The evolving landscape of integrative oncology models is focusing on patient-centred care and decision-making where supplementation is part of the larger discussion covering quality-of-life evaluation, symptoms-based management, and patient care (250). The translational potential of natural supplements in breast cancer advocates a new shift of paradigm for an evidence-based, personalized approach to supplementation that is mechanistically justified, clinically substantiated and integrated efficacy with standard treatment modalities, promising toward enhancing therapeutic effectiveness efficiency and patient survival in the care of breast cancer (251).

Recent convergence of artificial intelligence (AI) and big data analytics has presented unprecedented opportunities to address these translational barriers by large-scale, high-dimensional profiling of molecular, clinical, and lifestyle information (252). Machine learning algorithms with network-based computational models for example can be applied to search for response patterns among specific natural supplements in a broadly-based patient population, forecast synergy or antagonism of action with contemporary standard therapies and stratify patients according to molecular/metabolic signatures as the basis of a precision nutraceutical oncology. To find intriguing dietary supplements to study, safety signals, and biomarkers of effectiveness, artificial intelligence (AI)-enabled natural language processing (NLP) can help with structured mining of published literature, real-world data from EHRs and clinical trial databases, patient reported outcomes, and other sources (253). In addition, multi-omics big data integration genomic, transcriptomic, proteomic, metabolomic enables deeper insights into human variability in supplement metabolism and therapy efficacy that may realize personalized supplement strategies depending on tumour subtype, host biology under their life style (254). However, data standardization, validation of AI models, regulation for nutraceutical claims and incorporation into traditional oncology workflow remain challenging (255).

## 7 Limitations

Although increasing evidence indicate natural supplements as effective anticancer agents, several deficiencies impede their clinical translation and generalization. Small-sample-size-based or pilot-clinical trials used in this type of research makes studies underpowered. An additional issue relates to the quality, purity, and bioactive content of these supplements, which depend on the source of the plant, extraction method, or other regulatory aspects. One of the critical limitations is the absence of a standardized formula or content with an unknown dosing regimen. It is often found in earlier medicine research where concentrations tested on animal models are unattainable in the blood circulation of human patients, compromising physiological relevance. It applies to clinical trials that use different extraction forms, for example, curcumin-containing substances with unspecified amounts of curcuminoids that prevent establishing the equivalency of doses or the minimum therapeutic threshold Large, more centralized, and strictly controlled clinical trials are required in the future to provide more scientific evidence to assess conferred benefit and a more secure way for adding these natural supplements to evidence-based treatment options for breast cancer.

## 8 Conclusion

Natural products use in breast cancer therapy is an emerging field that holds promise for treatment as an alternative medicine. Although available evidence demonstrates their ability to increase treatment effectivity, reduce side effects of treatments and improve patient quality-life, the way to clinical implementation is challenging. Inspite of promising preclinical and observational data, their clinical translation remains limited due to challenges in bioavailability, lack of standardization, and possible herb–drug interactions. However, these challenges need to be overcome by a translational frame that includes pharmacogenomic profiling and nanotechnology-based delivery systems in future research. By defining molecular subtypes of disease and providing the relevant mix of personalized and individualized therapy, integrative oncology could be redefined in the form of personalized supplement regimens based on patient defined risk factors. The next generation of breast cancer treatment may combine traditional and complementary models of care within cost-effective, patient-centred, evidence-based frameworks for comprehensive wellbeing. Future research should focus on large, multicentre trials, optimization of pharmacokinetics and comprehensive safety profiling to substantiate the efficiency in various patient populations and molecular subgroups.
